# High-Throughput, Temporal and Dose Dependent, Effect of Vitamins and Minerals on Chondrogenesis

**DOI:** 10.3389/fcell.2020.00092

**Published:** 2020-02-25

**Authors:** James E. Dennis, Taylor Splawn, Thomas J. Kean

**Affiliations:** ^1^Department of Orthopedic Surgery, Baylor College of Medicine, Houston, TX, United States; ^2^Biionix Cluster, Internal Medicine, College of Medicine, University of Central Florida, Orlando, FL, United States

**Keywords:** cartilage tissue engineering, defined chondrogenic media, high-throughput chondrogenesis, high-throughput organoid, *in vitro* chondrogenesis, defined media

## Abstract

Tissue engineered hyaline cartilage is plagued by poor mechanical properties largely due to inadequate type II collagen expression. Of note, commonly used defined chondrogenic media lack 14 vitamins and minerals, some of which are implicated in chondrogenesis. Type II collagen promoter-driven *Gaussia* luciferase was transfected into ATDC5 cells to create a chondrogenic cell with a secreted-reporter. The reporter cells were used in an aggregate-based chondrogenic culture model to develop a high-throughput analytic platform. This high-throughput platform was used to assess the effect of vitamins and minerals, alone and in combination with TGFβ1, on *COL2A1* promoter-driven expression. Significant combinatorial effects between vitamins, minerals, and TGFβ1 in terms of *COL2A1* promoter-driven expression and metabolism were discovered. An “optimal” continual supplement of copper and vitamin K in the presence of TGFβ1 gave a 2.5-fold increase in *COL2A1* promoter-driven expression over TGFβ1 supplemented media alone in ATDC5 cells.

## Introduction

Osteoarthritis is a leading cause of disability with annual United States healthcare costs in excess of $500 billion and is the impetus for this study (Helmick et al., 2008; Centers for disease control and prevention, 2009). This laboratory has investigated media formulations and expansion surfaces to optimize cartilage formation for multiple applications (Henderson et al., 2007, 2010; Weidenbecher et al., 2007, 2008; Kean and Dennis, 2015; Kean et al., 2016; Dennis et al., 2018). Supplements include BMP (Mounts et al., 2012) and thyroxine (Whitney et al., 2018); nevertheless, engineered cartilage invariably contained type II collagen at levels far lower than native (Whitney et al., 2014), as is the scourge of the field. Current methods used to optimize medium conditions are tedious and inefficient. The goal of this study was to streamline the method to screen for optimal culture conditions using a high-throughput assay of a *Gaussia* luciferase reporter system linked to a type II collagen promoter. This initial study was applied to the optimization of the base medium components used to differentiate chondrocytes, using ATDC5 cells as a model system.

Multiple laboratories have focused on methods to engineer functional cartilage for various applications. In many instances, the mechanical properties fall short of that of native cartilage tissue. One major concern is that the diminished biomechanical properties of engineered cartilage are predominantly a result of insufficient production of type II collagen, which is the most abundant molecule in cartilage after water (Sophia Fox et al., 2009). Results in our laboratory have shown type II collagen content to be approximately 20% of that in native tissue (Whitney et al., 2018). Other laboratories have reported similar results, such as those of Sato et al. (2017) who showed type II collagen levels from cultured human MSCS at 20% that of native tissue, even after enhancement by the addition of epigallocatechin-3-gallate, which doubled the type II collagen output. Similar results were shown by Gemmiti and Guldberg (2006) where collagen content of bioreactor-grown bovine chondrocytes was 25% that of native collagen content, and in a study by Shahin and Doran (2011) where type II collagen levels were 18% that of native tissue. Other studies have shown collagen levels at less than 10% of native tissue (Mahmoudifar and Doran, 2010; Tian et al., 2013).

Many researchers, including ourselves (Kean and Dennis, 2015; Kean et al., 2016; Dennis et al., 2018; Whitney et al., 2018), use serum-free chondrogenic media to engineer cartilage *in vitro* (Johnstone et al., 1998). Upon examination of the composition of this medium, it was noted that several vitamins and minerals potentially relevant to chondrogenesis were absent (Table 1). To address this disparity and examine their potential effects on chondrogenesis, a high-throughput assay was developed based on a *COL2A1*-*Gaussia* luciferase reporter system using ATDC5 cells formatted as aggregates in 96-well plates. The dose responses of each vitamin and mineral was assessed over time. TGFβ1 was used as a positive control, as it has previously been reported to upregulate chondrogenesis in ATDC5s (Han et al., 2005) and other mammalian cells (Puetzer et al., 2010). Combinations of vitamins and minerals which showed positive increases in type II collagen promoter activity were tested in the presence of TGFβ1. This article shows proof of concept data for a high-throughput screen and establishes an improved chondrogenic medium for ATDC5 cells.

**TABLE 1:** Vitamins and minerals absent in DMEM [and therefore defined chondrogenic media (Kean and Dennis, 2015)].

Component	Role in chondrogenesis	Normal range^1^	References
Chromium	Unknown, has a role in insulin/glucose metabolism	0.3–28 μg/L	Finch, 2015
Cobalt	Unknown, required for vitamin B12 and other enzymes	0.0–0.9 μg/L	Mobasheri et al., 2006; MayoClinic, 2016
Copper	Part of lysyl oxidase complex – collagen crosslinking	90–670 μg/L	Makris et al., 2013a; Finch, 2015
Iodine	Deficiency causes cartilage defects	40–92 μg/L	Ren et al., 2007; MayoClinic, 2016
Manganese	Deficiency results in skeletal abnormalities	0.6–12 μg/L	Finch, 2015
Molybdenum	Unknown	0.3–2.0 μg/L	Sardesai, 1993; MayoClinic, 2016
α-linolenic acid	Unknown, precursor of phospholipid membrane	2.8–54 mg/L	Connor, 1999; MayoClinic, 2016
Thyroxine^2^	Enhances and stimulates terminal differentiation	9–17 ng/L	Mello and Tuan, 2006; MayoClinic, 2016; Whitney et al., 2018
Vitamin A	Shown to inhibit chondrogenesis	113–780 μg/L	Pacifici et al., 1980; Lind et al., 2013; Masuda et al., 2015; MayoClinic, 2016
Vitamin B7	Unknown	57–3004 ng/L	Ren et al., 2007; MayoClinic, 2016
Vitamin B12	Unknown, deficiency causes growth retardation	180–914 ng/L	Stabler, 2013; MayoClinic, 2016
Vitamin D	Implicated in chondrogenesis	24–86 ng/L	Tsonis, 1991; MayoClinic, 2016
Vitamin E	Unknown	3.8–18.4 mg/L	Wluka et al., 2002; MayoClinic, 2016
Vitamin K	Possible role in early chondrogenesis	0.1–2.2 μg/L	Barone et al., 1991; Booth, 1997; MayoClinic, 2016
Zinc	Stimulates chondrocyte growth and collagen production	0.6–1.2 mg/L	Koyano et al., 1996; Litchfield et al., 1998

*^1^Normal reference range in blood or serum; synovial fluid is not regularly assessed for these components. Defined chondrogenic media is supplemented with vitamin C and ITS + Premix which contains selenium and linoleic acid, thus providing three essential vitamins and minerals absent in DMEM. ^2^Thyroxine, although a growth factor, was included because of our previous study showing upregulation of type II collagen (Whitney et al., 2018).*

Somewhat surprisingly, the common cell culture medium Dulbecco’s Modified Eagle’s Medium (DMEM) used in cell culture of chondrocytes (and other cells) lacks several vitamins and minerals that are defined as essential (see Supplementary Table S1 for common media comparison). It is likely that some or all of these essential components are provided by the supplementation of fetal bovine serum during expansion, but these components are clearly absent during chondrogenic culture using defined medium (Johnstone et al., 1998; Kean and Dennis, 2015). Chondrogenic media compositions have evolved to defined media from that originally reported by Ahrens et al. (1977) to a reduced serum medium described by Ballock and Reddi (1994), then to a serum-free defined medium published by Johnstone et al. (1998); our current chondrogenic medium is an adaptation of the last (Kean et al., 2016). Table 1 shows the essential vitamins and minerals lacking in DMEM, a base medium commonly used in chondrogenic differentiation by us and others. Indeed, Makris et al. (2013b) found that copper supplementation of this base medium enhances collagen crosslinking and mechanical properties in tissue engineered cartilage. Because synovial fluid is rarely sampled from healthy patients, tests in our studies were based on the normal range of vitamins and minerals found in serum. Yazar et al. (2005) found mean zinc and copper levels of 114.1 and 280.8 μg/L, respectively in the synovial fluid. Niedermeier et al. (1962) in a semi-quantitative assay found mean copper, chromium and manganese levels of 210, 17, and 21 μg/L, respectively from synovial fluid extracted from cadaver knees. These data support the use of the normal serum range in our studies. To investigate this and other media supplements on chondrogenic differentiation, we assayed for *Gaussia* luciferase levels in the conditioned medium of ATDC5 cells, a commonly used chondrocyte cell line derived from mouse teratocarcinoma AT805, transduced with lentiviral *Gaussia* luciferase under the control of the *COL2A1* promoter (col2gLuc; HPRM22364-LvPG02, GeneCopoeia, Inc.). As a surrogate for testing RNA expression of type II collagen, a destructive assay, a secreted *Gaussia* luciferase assay was developed. Further, the use of the secreted form of luciferase allows for the temporal assessment of *COL2A1* promoter-driven expression in the conditioned medium of aggregate cultures.

## Results

### Pseudolentiviral Particle Production, Infection, and Selection

Pseudolentiviral particles of control (eGFP) and col2gLuc were successfully separately produced and used to infect ATDC5 cells. Dilution titration of infected cells with eGFP determined a multiplicity of infection (MOI) of 29. Calculation of infectious particle concentration by qPCR determined this concentration to be approximately the same for col2gLuc in terms of viral particles per μL (1.9 × 10^7^ and 1.6 × 10^7^ for eGFP and col2gLuc, respectively), this equates to an approximate MOI of 25 for the col2gLuc studies.

### Basal ATDC5 Culture Optimization

**FIGURE 1:**
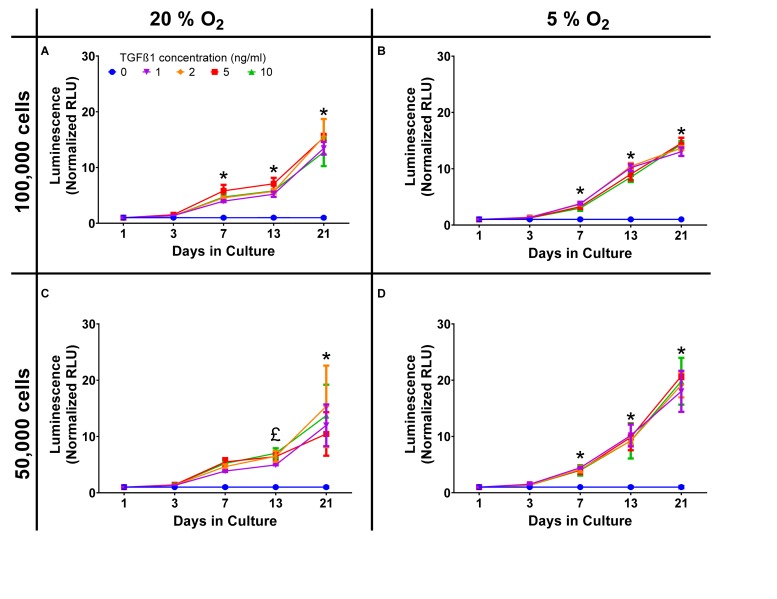
Oxygen and cell density response. Dose response curves, normalized to the control of 0 ng/ml TGFβ1 in chondrogenic media, were constructed and show a time dependent increase in luminescence but with similar responses in terms of both cell number and oxygen tension. **(A)** 20% O_2_, 100,000 cells; **(B)** 5% O_2_, 100,000 cells; **(C)** 20% O_2_, 50,000 cells; **(D)** 5% O_2_, 50,000 cells. * = all concentrations significantly greater than basal media, £ = concentrations ≥ 2 ng/ml TGFβ1 significantly greater than basal media (*p* < 0.05).

Standard cell culture conditions are generally performed at supraphysiological oxygen tensions, for chondrocytes and tissue engineered cartilage this can be particularly detrimental in terms of extracellular matrix production. In order to demonstrate that there were limited effects on *Gaussia* luciferase a comparison between standard (20%) oxygen tension and physiological (5%) oxygen tension experiment was performed. In initial experiments, chondrocytes were plated in high-density pellet cultures at 50,000 and 100,000 cells/pellet at both 20% oxygen and 5% oxygen [physioxia (Kean and Dennis, 2015; Kean et al., 2016)], with similar *Gaussia* luciferase responses to TGFβ1 in each (Figure 1). Data were averaged and the *z*-factor calculated according to the formula described by Zhang et al. (1999).

$$\frac{\left(\mathrm{PC}\mathrm{average}\left[5\text{ng}/\text{mL}\right]-3\times \text{PC}\text{S}.\text{D}.\right)-\left(\mathrm{NC}\mathrm{average}\left[0\text{ng}/\text{mL}\right]+3\times \text{NC}\text{S}.\text{D}.\right)}{\left(\mathrm{PC}\,\mathrm{average}-\mathrm{NC}\,\mathrm{average}\right)}$$

where PC, positive control and NC, negative control.

For day 21 the *z*′ factor for a 50,000 cell pellet was 0.89, which is regarded as very good, validating the assay as having sufficient sensitivity for high-throughput applications. Luminescent decay was assessed over 50 min and the half-life of positive (10 ng/mL) and negative (0 ng/mL) samples were similar; 27.8 and 33.1 min, respectively. As significant signal to noise was achieved using a lower number of cells, the remaining experiments were all conducted using 50,000 cell aggregates in physioxia (5% O_2_) and luminescence read within 10 min of adding stabilized substrate. Correlation between the expression of *COL2A1* promoter-driven *Gaussia* luciferase and type II collagen expression was established by qPCR with an *R*^2^ value of 0.85 (Day 3, Supplementary Data S1).

### TGFβ1 Dose Response

**FIGURE 2:**
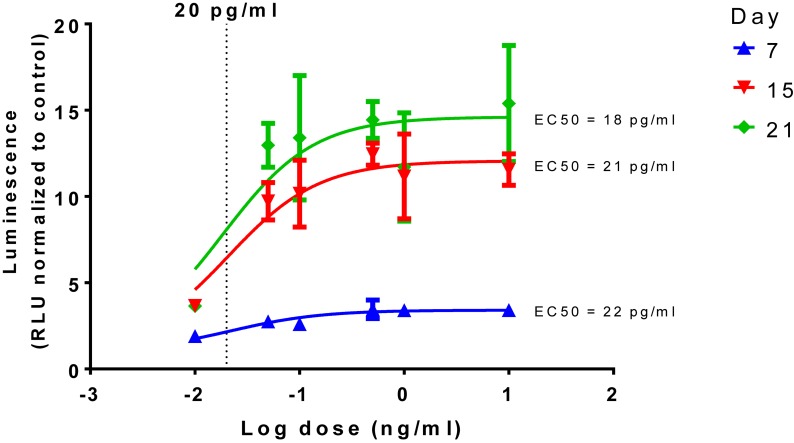
Time and dose dependent effect of TGFβ1 on chondrogenesis. Dose response curves, normalized to the control of 0 ng/mL in chondrogenic media, were constructed and show a time dependent increase in luminescence but with similar EC50s, a dotted line is shown to show 20 pg/mL in the log scale.

The dose response curve of TGFβ1, a known inducer of chondrogenesis, was determined along with the 50% effective concentrations (EC50) calculated as 18, 21, and 22 pg/mL for days 7, 15, and 21, respectively (Figure 2).

### Vitamin and Mineral Dose Response in Basal Media

Vitamins and minerals were assessed at varying concentrations based on normal serum concentrations with amounts ranging from 0.01 to 1000% of the maximal serum concentration. Both dose- and time dependent effects on *COL2A1* promoter-driven expression were evident (Table 2 and Supplementary Data S2). Typical dose response curves, such as that found for TGFβ1 were not evident; as might be expected for a compound acting in multiple systems as a catalyst/co-factor. All supplements except a-linolenic acid had increased expression at the higher doses at the 2 weeks time point. a-linolenic acid treatment showed increased expression at low doses during the early portion of the experiment which became detrimental at later stages. This decrease at day 21 might indicate an earlier maturation of the tissue. A small, but significant increase in promoter-driven luciferase expression was seen in most cases at one or more doses. A consistent increase over basal expression was seen with chromium (2.8 μg/L; 10% serum max) and vitamin E (1.8–9.2 mg/L; 10–50% serum max) throughout. Vitamin A, widely regarded as detrimental to chondrogenesis, was actually stimulatory to type II collagen promoter-driven expression at the lowest dose tested (78 ng/L; 0.01% serum max) except for the last time point which had returned to basal expression levels.

**TABLE 2:** Temporal effect of vitamins and minerals on chondrogenesis in basal media.

Component	Dose (% of maximal serum concentration^‡^)	Fold change vs. basal medium^£^			
		
		Week			
		
		0.4	1	2	3
Chromium	2.8 μg/L (10)	1.73*	2.05*	2.29*	2.0*8
Cobalt	9.00 μg/L (1000)	1.17	1.45	4.65*	1.03
Copper	6.70 mg/L (1000)	0.78	0.68	8.61*	0.16*
Iodine	920 μg/L (1000)	1.19*	1.37	4.79*	0.95
Manganese	60 μg/L (500)	1.06	1.04	4.33*	0.99
Molybdenum	20 μg/L (1000)	1.25	1.34	4.69*	1.11
α-linolenic acid	5.4 μg/L (0.1)	1.51*	1.37*	1.02	0.82
Vitamin A	0.078 μg/L (0.01)	1.94*	2.22*	3.23*	1.06
Vitamin B7	15.02 μg/L (500)	1.63*	1.68*	1.22	1.36
Vitamin B12	91.4 ng/L (10)	1.68*	1.70*	1.63*	1.37
Vitamin D	8.6 ng/L (10)	1.31*	1.02	0.86	0.68*
Vitamin E	18.4 mg/L (100)	1.51*	1.56*	1.44*	1.16
Vitamin K	22 μg/L (1000)	0.97	0.94	5.68*	1.04
Zinc	6.0 mg/L (500)	1.27	1.54	1.10	1.51*

^‡^See Table 1 for normal reference range in blood or serum; synovial fluid is not regularly assessed for these components; defined chondrogenic media is supplemented with vitamin C and ITS + Premix which contains selenium and linoleic acid, which are absent in DMEM. *Significantly different vs. basal medium, two-way ANOVA *p* < 0.05 Sidak’s multiple comparison test. £Fold change of component supplemented luminescence relative to basal control luminescence of Gaussia luciferase from conditioned media.

### Vitamin and Mineral Combinatorial Effects

**FIGURE 3:**
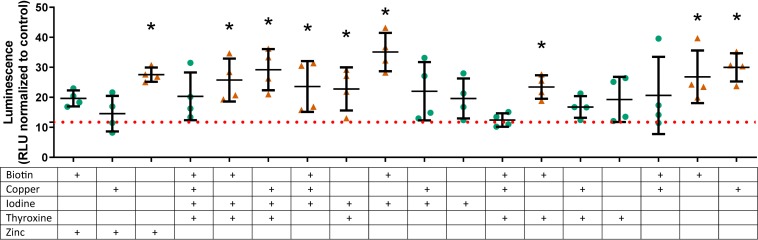
Type II collagen promoter-driven expression with various vitamin and mineral combinations in the presence of TGFβ1 on day 21. Vitamins and minerals (biotin, copper, iodine, thyroxine, and zinc) which had been identified as promoting *COL2A1* promoter-driven expression in the initial screen were tested in combination at their individually optimized concentrations (3, 670, 0.92, 20, and 840 μg/L, respectively) in the presence of TGFβ1 at 1 ng/mL. The values are normalized to the plate control (basal media). Green circles (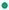) indicate values that are significantly greater than those for basal media alone, and the dashed red line indicates the luminescence achieved with TGFβ1 supplementation at 1 ng/mL. An orange triangle (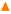) and an * indicate values that were discovered different, with false discovery rate set at 1%.

A selection of those factors that had overall positive effects on the relative luminescence of the conditioned media (vitamin B7, copper, iodine, thyroxine, and zinc) were tested at their optimal individual concentration in basal medium and in combination with TGFβ1 (1 ng/mL). These six factors gave 64 conditions; 53 of which were discovered to be significantly different vs. the basal medium, with 46 being increased and 7 being decreased at day 21 (Supplementary Data S3). An abbreviated selection of conditions in the presence of TGFβ1 is shown in Figure 3 (18 of 64); several conditions in combination with TGFβ1 are greater than TGFβ1-supplemented basal medium alone (15 of 31; Figure 3 and Supplementary Data S3). The results showed several interesting temporal patterns.

**FIGURE 4:**
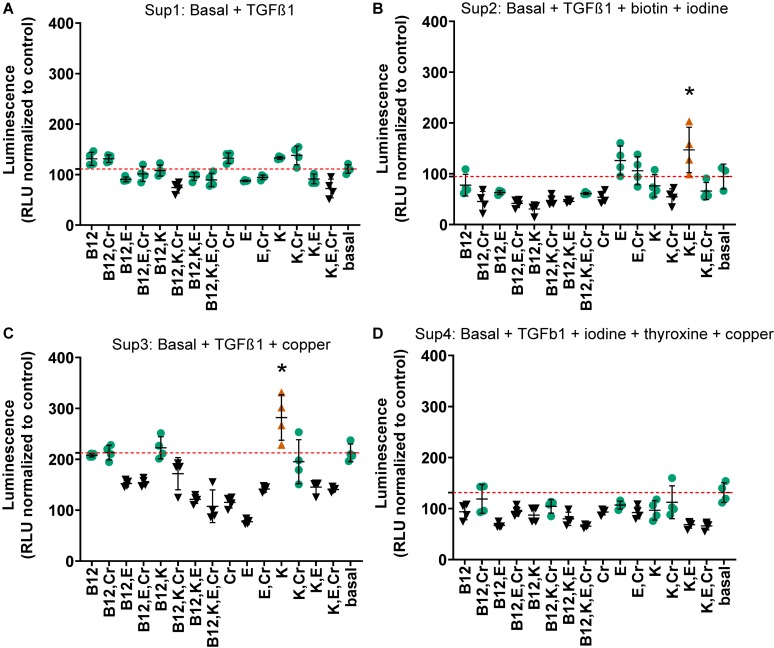
Combinations of chromium, vitamins B12, E, and K in supplemented media at day 21. Vitamins and minerals (chromium, vitamins B12, E, and K) which had been identified as promoting *COL2A1* promoter-driven expression in the initial screen were tested alone and in combination in supplemented medias 1 **(A)** Basal media + TGFβ1; 2 **(B)** Basal + TGFβ1 + biotin + iodine; 3 **(C)** Basal + TGFβ1 + copper; 4 **(D)** Basal + TGFβ1 + iodine + thyroxine + copper. Vitamins and minerals were supplemented at their individually optimized concentration in basal media (2.8 μg/L, 91.4 ng/L, 1.84 μg/L, and 2.2 μg/L, respectively). The values are normalized to the plate control (basal media); the red dashed red line indicates the luminescence achieved with that particular supplemented media alone. Green circles indicate values that are not significantly different to supplemented media (red dashed line), all are greater than basal media. Conditions that were statistically greater than their respective supplemented media are represented by an orange triangle (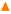) and an *, black symbols (▼) indicate that those values were lower than the respective supplemented media. *n* ≥ 3 ± S.D.

Due to the prohibitively large number of combinations with the remaining 10 factors (1023 conditions), the three highest performing combinations from the first combination experiments, the results of which are shown in Figure 3, were further supplemented with combinations of vitamins B12, E, K, and chromium. Each of these vitamins and minerals had been determined to effect an increase in relative luminescence over basal media individually. Chromium, vitamins B12, K, and E were supplemented at their individually determined optimal concentrations in basal media (2.8 μg/L, 91.4 ng/L, 1.84 μg/L, and 2.2 μg/L, respectively). The three highest performing combinations from the previous experiment were used for this phase of the study and the TGFβ1 supplemented basal media. These media are termed Sup 1 (basal + TGFβ1); Sup 2 = Sup 1 + iodine + biotin; Sup 3 = Sup 1 + copper; and Sup 4 = Sup 1 + iodine + thyroxine + copper. This resulted in assessment of an additional 60 conditions. In this set of experiments, Sup 3 was higher than Sup 1, as was shown previously in Figure 4, and several combinations were lower than the supplemented media in this and Sup 4 comparisons (Figure 4 and Supplementary Data S4). Although all combinations significantly increased *COL2A1* promoter-driven expression over that of basal medium, only vitamin K significantly increased luminescence above TGFβ1- and copper-supplemented basal medium (2.5-fold vs. TGFβ1 alone; Sup 3; Figure 4C and Supplementary Data S4). However, several combinations showed luminescence levels lower than baseline supplemented media (Sup 3 and Sup 4).

### Dose Response to Vitamin and Mineral Supplementation in the Presence of TGFβ1

**FIGURE 5:**
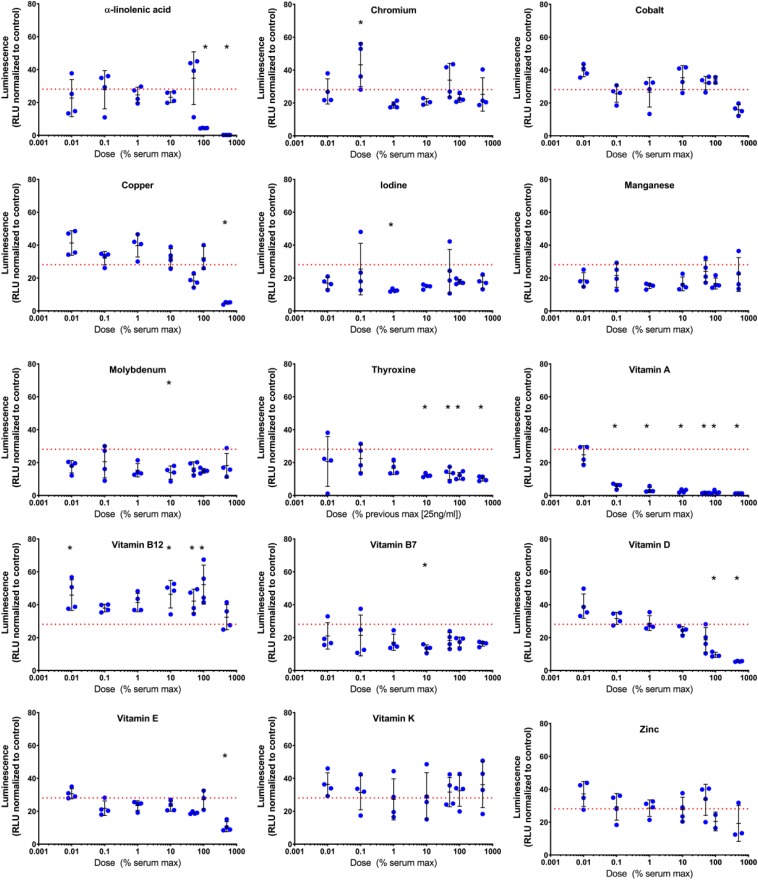
Day 21 evaluation of *COL2A1* promoter-driven expression in vitamin and mineral supplemented TGFβ1 supplemented basal medium. Conditioned media was sampled at day 21 and assessed for luminescence, and normalized against the plate control basal medium. The red dashed line indicates the luminescence of the TGFβ1 supplemented basal medium. The symbol “*” significantly different vs. TGFβ1 supplemented basal medium, two-way ANOVA *p* < 0.05 Sidak’s multiple comparison test. Blue circles represent each data point.

Given the complex responses seen with combinations in the presence of TGFβ1, vitamin and mineral dose responses (0.01–500% serum max) were further tested in the presence of TGFβ1 (1 ng/mL). Both time and dose responses were evident (Table 3, Figure 5 and Supplementary Data S5). Conditioned medium was collected on days 3, 7, 15, and 21 and assayed for *Gaussia* luciferase expression to evaluate the temporal effects of different additives. The results were expressed as the fold change relative to time-matched basal medium controls (Table 3).

**TABLE 3:** Dose and temporal effect of vitamins and minerals in the presence of TGFβ1 on chondrogenesis.

Component	Dose (% of maximal serum concentration^‡^)	Fold change vs. basal medium£			
		
		Day			
		
		3	7	15	21
Chromium	28 ng/L (0.1)*	2.2	8.2	29.1	43.3
Cobalt	90 pg/L (0.01)*	1.8	5.0	22.1	39.5
Copper	67 ng/L (0.01)*	1.9	5.6	23.5	41.3
Iodine	92 ng/L (0.1)	2.5	5.4	17.4	25.4
Manganese	12 ng/L (0.1)	1.8	5.8	16.4	21.6
Molybdenum	2 ng/L (0.1)	1.7	5.2	15.3	20.5
α-linolenic acid	27 mg/L (50)	2.0	7.1	27.1	34.8
Thyroxine	25 ng/L (147)*	1.7	6.0	18.1	22.6
Vitamin A	78 ng/L (0.01)	2.3	5.5	15.7	24.8
Vitamin B7	3 ng/L (0.1)	1.6	5.0	15.5	21.4
Vitamin B12	914 ng/L (100)*	1.9	7.2	35.5	52.2
Vitamin D	86 pg/L (0.01)*	1.7	5.1	20.0	39.2
Vitamin E	18.4 mg/L (100)	1.6	6.6	21.7	27.1
Vitamin K	11.0 μg/L (500)*	2.6	8.1	24.7	36.2
Zinc	0.12 μg/L (0.01)*	1.7	5.0	19.0	37.2
TGFβ1 medium	1 μg/L (956^t^)	1.7	5.3	16.3	28.1

^‡^See Table 1 for normal reference range in blood or serum. ^*t*^Maximum normal adult serum TGFβ1 is 104.6 pg/mL (Tas et al., 2014), this experiment used 1 ng/mL. Synovial fluid has ∼1.8 ng/mL latent TGFβ1 and <0.05 ng/mL active TGFβ1 (Albro et al., 2012); defined chondrogenic media is supplemented with vitamin C and ITS + Premix which contains selenium and linoleic acid, which are absent in DMEM. *Slope or intercept of linear regression significantly different compared to TGFβ1 supplemented basal medium *p* < 0.05. £Fold change of component supplemented luminescence relative to basal control luminescence of *Gaussia* luciferase from conditioned media.

**FIGURE 6:**
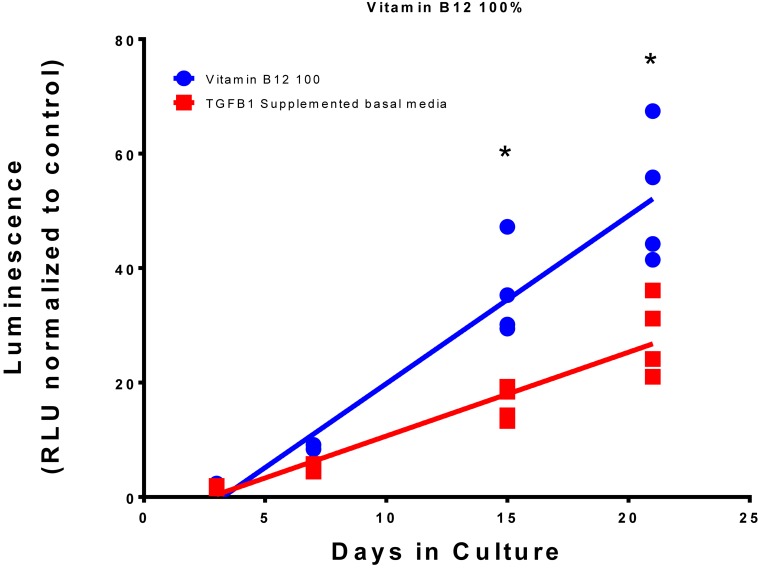
Comparison of Vitamin B12 and TGFβ1 supplemented medium with TGFβ1 supplemented basal medium. Type II collagen promoter-driven luminescence was assessed from the conditioned media and normalized to the plate basal medium control. Lines indicate a linear regression; symbols indicate individual data points. The symbol “*” significant difference vs. TGFβ1 supplemented basal medium; two-way ANOVA *p* < 0.05 Sidak’s multiple comparison test.

Summation of the fold change across the whole experiment to give area under the curve analysis identified 40 conditions that were greater than the TGFβ1 supplemented medium. The top 10 conditions that gave greater expression over the whole experiment are shown in Table 4 with an example plot in Figure 6 (the remaining comparisons are shown in Supplementary Data S7).

**TABLE 4:** Overall dose and time response.

Condition and dose (%)	Area under curve
Vitamin B12 (100)	434.3
Chromium (0.1)	369.1
Vitamin B12 (10)	355.2
Vitamin B12 (0.01)	348.2
Vitamin B12 (50)	341.9
a-linolenic acid (50)	323
Vitamin K (500)	317.3
Copper (0.01)	308.1
Chromium (50)	303.8
Vitamin B12 (1)	297.7
BR + TGFβ1 (5000)	215.4

**FIGURE 7:**
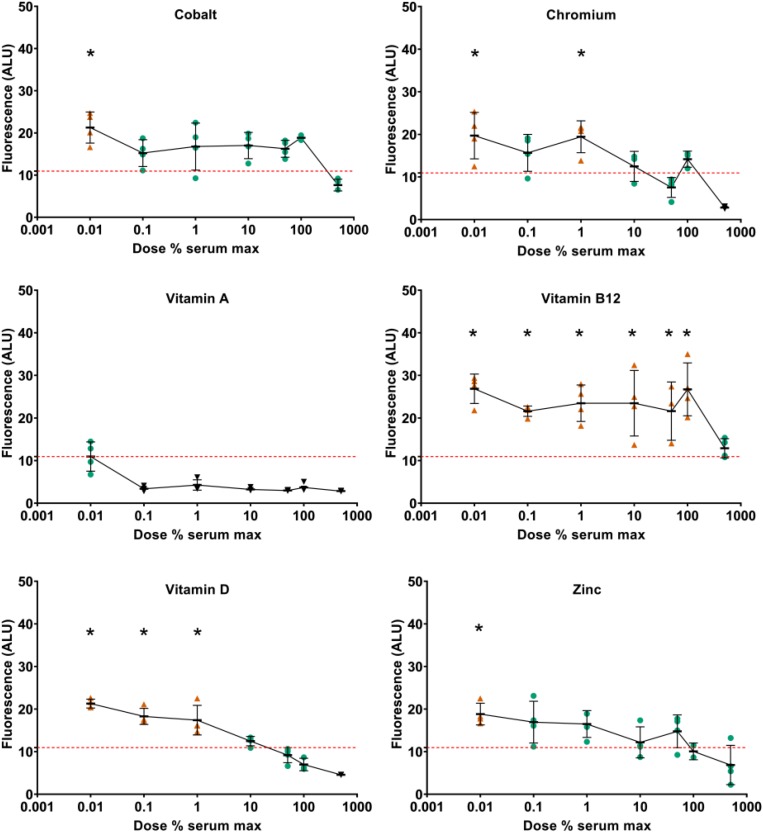
Effect of vitamin and mineral addition to TGFβ1 supplemented medium on metabolic activity. Cells were treated with resazurin, media was sampled at day 21 and assessed for fluorescence. All data were normalized against the conditioned media from pellets treated with basal chondrogenic media. The red dashed line indicates the fluorescence of the TGFβ1 supplemented basal medium. An orange triangle (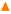) and an * indicate greater than TGFβ1 supplemented basal medium; black triangles (▼) indicate lower vs. TGFβ1 supplemented basal medium, one-way ANOVA *p* < 0.05 Dunnett’s multiple comparison test. Conditions that were similar to TGFβ1 supplemented basal media are represented by a green circle (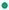).

To assess the effects of supplementation on metabolic activity, resazurin assays were performed at day 21. At day 21, increased metabolic activity was detected at low doses of cobalt, copper, vitamin D, vitamin E, and zinc (Figure 7 and Supplementary Data S6). Decreased metabolic activity was seen at high doses of copper and vitamin D. Vitamin A was predominantly inhibitory and vitamin B12 predominantly stimulatory. When type II collagen promoter-driven luciferase expression was normalized against resazurin metabolism, none of the components tested showed increased expression at day 21 compared to TGFβ1 supplemented control except iodine at 92 μg/L. Indicating that, at this time point, the effect on type II collagen promoter-driven luciferase was predominantly or exclusively linked to metabolic activity (Supplementary Data S10).

## Materials and Methods

### ATDC5 Cell Culture and Transformation

ATDC5 cells (a kind gift of Dr. Florent Elefteriou, Baylor College of Medicine) were grown in DMEM-LG (HyClone) containing 5% FBS (Atlanta Biologicals) and 1% penicillin/streptomycin (Gibco) at 37°C in a humidified atmosphere with 20% O_2_, 5% CO_2_. Cells were transformed with pseudolentiviral particles during log expansion phase at approximately 30% confluence. Concentrated lentivirus was diluted with growth medium and mixed 1:1 with polybrene (8 μg/mL; EMD Millipore) in Opti-MEM (Gibco), then applied to the cells. This concentration of polybrene was determined to have minimal effects on proliferation and significantly improved infection. Plates were incubated at 4°C for 10 min before transfer to 37°C (humidified atmosphere with 20% O_2_, 5% CO_2_) for 12 h. Media were then exchanged for growth medium and cells were grown to ∼90% confluence. Cells were then trypsinized and re-plated at 6000 cells/cm^2^. After allowing cells to adhere for 24 h, infected cells were selected by incubation in growth medium containing puromycin (2 μg/mL; Alfa Aesar) for 1 week. This concentration was determined to be the lowest concentration with >95% uninfected cell death on human mesenchymal stromal cells and chondrocytes (data not shown). Puromycin-selected cells were then grown in normal growth medium for no more than four passages before use in differentiation experiments.

### Lentiviral Construct

The collagen type II promoter (*COL2A1*)-driven, secreted *Gaussia* luciferase was custom ordered (HIV-based lentiviral third generation; HPRM22364-LvPG02, GeneCopoeia, Inc.). The promoter length is 1426 bp. This plasmid (col2gLuc; HPRM22364-LvPG02), the eGFP control (EX-EGFP-Lv105, GeneCopoeia, Inc.) the packaging plasmid (psPAX2) and the envelope plasmid (pMD2.G) were amplified in *Escherichia coli* (GCI-L3, GeneCopoeia, Inc.), and purified with a silica column (Qiagen Maxiprep). Plasmids (Supplementary Data S9) were confirmed by restriction digest and agarose gel electrophoresis. Pseudolentiviral particles were made in HEK-293Ta cells (GeneCopoeia, Inc.) by co-transfection of psPAX2, pMD2.G, and HPRM22364-LvPG02 using calcium phosphate precipitation (Dull et al., 1998). Pseudolentiviral particles were harvested from conditioned medium at 48 h and concentrated by centrifugation (10,000 RCF, 4°C, overnight). Infectious particle concentration was estimated by serial dilution of pseudolentiviral particles and flow cytometry assessment of eGFP positive cells produced in the same batch (Cellecta, 2018). The concentration of lentiviral particles used in these experiments was the most dilute concentration that resulted in >95% eGFP positive cells. Lentiviral particle concentration was further determined by qPCR assessment of lentiviral RNA according to the manufacturer’s instructions (Lenti-Pac Titration Kit; LT005, GeneCopoeia, Inc.).

### qPCR Correlation of COL2A1 Promoter-Driven Gaussia Luciferase With Type II Collagen Expression

Cells were lysed and RNA purified with guanidine based extraction RNA according to the manufacturer directions (Direct-zol RNA Miniprep, Zymo Research). To create cDNA, 750 ng of purified RNA was reverse transcribed according to the manufacturer directions (SuperScript^TM^ III Platinum, Invitrogen) using oligoDT primers. Quantification of gene expression was performed by qPCR using SYBR green (Power SYBR, Invitrogen) detection with the following primers: *Gaussia* luciferase F: 5′–ACG CTG CCA CAC CTA CGA–3′, R: 5′–CCT TGA ACC CAG GAA TCT CAG GAA–3′; murine type II collagen F: 5′–CCA ATG ATG TAG AGA TGA GGG C–3′, R: 5′–TGT GTT GTT TCA GGG TTC GGG–3′. *Gaussia* luciferase primer efficiency was calculated from a cDNA dilution curve at 100.4%, with an amplicon length of 92 bp and a melt temp of 81.5°C (CFX96 real time, Bio-Rad Laboratories, Inc.), no cross-reactivity was found with the mouse genome [Primer-Blast (Ye et al., 2012)]. Murine *Col2a1* primers amplify both variants [NM 031163.3 (variant 1) and NM 001113515.2 (variant 2)]; primer efficiency was calculated from a cDNA dilution curve at 93.6%, with an amplicon length of 243 bp and a melt temp of 81.5°C (CFX96 real time, Bio-Rad Laboratories, Inc.).

### Chondrogenic Differentiation

Cells were differentiated in pellet culture at 37°C in a humidified atmosphere with 5% O_2_, 5% CO_2_ (50,000–250,000 cells/pellet) in polypropylene 96-well plates (Phenix) in defined basal chondrogenic medium: DMEM-HG supplemented with 1% insulin, transferrin, selenium + premix; 130 mM ascorbate-2-phosphate, 2 mM GlutaMax, 1% sodium pyruvate, 1% MEM non-essential amino acids, 100 nM dexamethasone, 1.25 μg/mL fungizone, and 1% penicillin/streptomycin. This medium was further supplemented with vitamins and minerals (Table 5), thyroxine (Whitney et al., 2018) and recombinant human TGFβ1 (PeproTech). Medium sampling, mixing and exchange were conducted with a Tecan Freedom Evo fitted with a 96-head multi-channel aspirator (MCA) and 4-channel dispensing tips (DiTi). The pipetting profile for the MCA was set to aspirate at 10 μL/s and dispense at 500 μL/s. The pipetting profile for the DiTi was set to aspirate at 20, 100, and 150 μL/s for <15, 15–200, and 200–1000 μL respectively and to dispense at 600 μL/s for all volumes.

**TABLE 5:** Sources and stock solution preparation of vitamins and minerals.

Component	Format	Stock solution	Catalog #	Supplier
Chromium	CrCl_3_:6H_2_O	H_2_O	27096	Sigma-Aldrich
Cobalt	CoCl_2_:6H_2_O	H_2_O	C8661	Sigma-Aldrich
Copper	CuSO_4_:5H_2_O	H_2_O	C7631	Sigma-Aldrich
Iodine	NaI	H_2_O	A15480	Alfa Aesar
Manganese	MnCl_2_:4H_2_O	H_2_O	M3634	Sigma-Aldrich
Molybdenum	(NH_4_)_6_Mo_7_O_24_:4H_2_O	H_2_O	M1019	Sigma-Aldrich
α-linolenic acid	CH_3_(CH_2_CH=CH)_3_(CH_2_)_7_CO_2_H	Ethanol	L2376	Sigma-Aldrich
Thyroxine	3,3′,5-Triiodo-L-thyronine sodium salt	1M NaOH	T6397	Sigma-Aldrich
Vitamin A	Retinoic acid	Ethanol	44570	Alfa Aesar
Vitamin B7	D(+)Biotin	H_2_O	A14207	Alfa Aesar
Vitamin B12	Cyanocobalamin	H_2_O	V6629	Sigma-Aldrich
Vitamin D	Calcitriol	Ethanol	S1466	Selleck Chemicals
Vitamin E	(±)-A-Tocopherol	Ethanol	T3251	Sigma-Aldrich
Vitamin K	Menaquinone (K2)	Ethanol	47774	Sigma-Aldrich
Zinc	ZnSO_4_:7H_2_O	H_2_O	33399	Alfa Aesar

### Luciferase Assessment

Conditioned cell culture medium was sampled from plates (20 μl/well; MCA) at feeding and pipetted into a white 96-well plate (Greiner Bio-One) and assessed for luminescence using the stabilized *Gaussia* Luciferase Kit (New England Biolabs, Inc.; 50 μl of a 1:1 dilution with 18 MΩ water) and read in a plate reader (Tecan M200 PRO; 5 s 1 mm orbital mix, 45 s wait, then read 2 s/well, 20°C, no attenuation). All data were normalized against the conditioned media from pellets treated with basal chondrogenic media. Basal conditioned medium from cells was supplemented with vitamins and minerals to determine if there was any effect on the luciferase assay, none was seen (Supplementary Data S8).

### Metabolic Assay

Metabolic activity was assessed on day 21 using the resazurin assay (O’Brien et al., 2000; Schreiber-Brynzak et al., 2015). Resazurin (Sigma) was dissolved in phosphate buffered saline to give a 10× stock solution (500 μM), sterile filtered (0.2 μm) and aliquots made and frozen (−80°C). Frozen aliquots were thawed and diluted in phosphate buffered saline to 50 μM, 20 μL of this solution was then added to each well and incubated at 37°C for 6 h. Fluorescence was read (Tecan Infinite^®^ 200 PRO) with excitation at 530 nm and emission at 560 nm.

### Statistics

Statistics were performed using GraphPad Prism (GraphPad Software, Inc.). Comparisons of dose response over time were made using a two-way ANOVA with Sidak’s multiple comparison *post hoc* test. Comparisons between supplemented media (Sup 1–4) and additives were made using a two-way ANOVA with Sidak’s multiple comparison *post hoc* test. Comparisons between basal or TGFβ1 supplemented media with combinations were made using multiple *t*-tests with a false discovery rate method of Benjamini and Hochberg set at 1%. Comparisons of the metabolic dose response due to supplementation with vitamins and minerals were made using ANOVA with Dunnett’s multiple comparison *post hoc* test.

## Discussion

Tissue engineering of cartilage has the potential to ameliorate disability due to arthritis by (a) providing a better *in vitro* model for drug discovery and (b) providing a biological replacement eliminating the need for total joint arthroplasty. Without a permissive environment, i.e., one that contains all the vitamins and minerals that have been deemed essential, reflecting *in vivo* conditions, drug discovery and tissue engineering efforts are potentially hampered by inappropriate responses. This is exemplified in this study through the synergistic effect of vitamins, minerals, and TGFβ1 on *COL2A1* promoter activity (Figure 3). It is perhaps unsurprising that vitamins and minerals which are known to be essential for proper development have significant effects on *COL2A1* promoter-driven expression in this *in vitro* system. It is therefore surprising that they are omitted from common basal media; this work establishes that, while not all components promote *COL2A1* promoter-driven expression, they can still have an effect on chondrogenesis, such as through the stimulation or inhibition of metabolism. These complex temporal effects are to be expected given the broad range of enzymes, signaling pathways, and physiological processes that require these vitamins and minerals for their activity.

The goal of the present study was to streamline the methodology for optimizing *COL2A1* promoter-driven expression and, at the same time, to use the examination of vitamins and minerals missing from conventional defined media both as a proof of concept and as a much needed inquiry. To accomplish that goal, an aggregate chondrogenesis assay (Yoo et al., 1998) that had been modified for a 96-well format (Penick et al., 2005) was applied as a means to use a high-throughput cartilage tissue-based assay to screen chondrocytes transduced with a *Gaussia* luciferase reporter driven by a type II collagen promoter. This has resulted in significant insights into the role of vitamins and minerals on chondrocyte metabolism. There is a clear interaction between TGFβ1 and some vitamins and minerals (copper, vitamin A). However, even at our reduced TGFβ1 concentration of 1 ng/mL, one-tenth of that commonly used but still ∼500-times the EC_50_ and ∼10-times normal serum concentration, we may have masked some potential stimulators.

To date, only a few attempts at developing high-throughput assays for chondrogenesis have been reported. Greco et al. (2011) developed what they termed a “medium-throughput” assay that used the human C-28/12 chondrocyte cell line where cells were seeded as 1.0 × 10^4^ micromasses in 24-well plates; terminal assays were used to determine gene expression and GAG content. Another high-throughput assay, using only 1.0 × 10^4^ cells per well, was used to screen for factors influencing mesenchymal stem cell chondrogenesis (Huang et al., 2008) where, again, terminal assays were used to assess gene expression and GAG content. A similar aggregate assay was also described for adipose-derived stem cells where 2.0–5.0 × 10^5^ cells per well of a 96-well plates were cultured for several weeks and then assayed for GAG content as a terminal assay (Abu-Hakmeh and Wan, 2014). Another study used ATDC5 cells in a high-throughput assay to test a library of factors for their effect on total collagen production (Le et al., 2015). In their study, ATDC5 cells were plated at 5.0 × 10^3^ cells per well and allowed to expand for 3 days and, after 6 days, were assayed for collagen content using a fluorescent collagen-binding probe (CN35-AF488) to measure collagen content. In all of these cases, the assays were conducted as terminal assays, that is, at the end of the incubation period. One of the strengths of the methodology shown here is that the assays are conducted on conditioned medium, allowing for a complete temporal assessment of the different factors, and combinations of factors, over time. Temporal chondrogenesis and osteogenesis have been studied by others with Correa et al. (2018) investigating Sox9, aggrecan, and osteocalcin in mesenchymal stem cells.

While the addition of components to media formulations makes their composition more complex, we would argue that, in order to model the effect of any *in vivo* manipulation, a permissive environment, i.e., one that contains factors essential for normal cell metabolism is necessary. Given that: (1) cells are relatively efficient in recycling many of their components, (2) *in vitro* systems are an imperfect model, and (3) the conditions that exist within a developing joint are relatively unknown, our current suggested “optimal” medium for chondrogenesis is: DMEM-HG supplemented with 1% insulin, transferrin, selenium + premix; 130 mM ascorbate-2-phosphate, 2 mM GlutaMax, 1% sodium pyruvate, 1% MEM non-essential amino acids, 100 nM dexamethasone, 1.25 μg/mL fungizone, 1% penicillin/streptomycin, 1 ng/mL TGFβ1, 670 ng/mL copper, 2.2 ng/mL vitamin K (highest yield, Figure 4). However, based on the apparent shift around day 15 both in this work and that shown by Huynh et al. (2018) there is potential for a media switch around this time. In addition, we consider that a permissive medium solution would contain other vitamins and minerals at 1/100th–1/1000th of their serum max, potentially allowing somewhat of a “normal” response to other manipulations such as mechanical stimulation (Nazempour et al., 2016). The proposed supplements to chondrogenic medium are shown in Table 6 for the two time periods 0–15 and 16–21 days based on their ability to consistently upregulate type II collagen promoter-driven *Gaussia* luciferase expression without compromising metabolic activity. Select vitamins and minerals were removed from the latter stage due to their correlation with decreased *COL2A1* promoter-driven expression after 15 days; this may be because the tissue is more mature, and they are recycled more efficiently and/or used less. The fat soluble vitamins A, D, E, K, and a-linolenic acid should be combined with ITS + premix as a stock solution as they will bind to the bovine serum albumin in that solution. These suggested media need further investigation in this and other systems, current work is focusing on the application of this system to primary chondrocytes.

**TABLE 6:** Proposed supplements to defined chondrogenic media.

Component	Final media concentration	Day 0–15	Day 16–21
α-linolenic acid	0.54 mg/L	✓	✓
Chromium	0.28 μg/L	✓	✓
Cobalt	90 pg/L	✓	✓
Copper	6.7 μg/L	✓	✓
Iodine	92 ng/L	✓	✓
Manganese	12 ng/L	✓	×
Molybdenum	0.2 ng/L	✓	×
Thyroxine	25 pg/L	✓	×
Vitamin A	78 ng/L	✓	×
Vitamin B7	3 ng/L	✓	×
Vitamin B12	914 ng/L	✓	✓
Vitamin D	8.6 pg/L	✓	✓
Vitamin E	18.4 mg/L	✓	✓
Vitamin K	11 μg/L	✓	✓
Zinc	120 ng/L	✓	✓

## Conclusion

A high-throughput amenable, temporal surrogate chondrogenesis assay has been developed in a model chondrocyte cell line, ATDC5. Medium supplementation with vitamins and minerals resulted in changes in *COL2A1* promoter-driven expression and metabolic activity dependent on both time and concentration.

## Summary

Current defined chondrogenic culture media lack several vitamins and minerals. Type II collagen is the quintessential marker of articular hyaline cartilage, and is commonly deficient in engineered tissue. A type II collagen promoter-driven secreted luciferase construct has been transduced into ATDC5 cells and used to assess vitamin and mineral effects on chondrogenesis in a high-throughput format.

## Data Availability Statement

All datasets generated for this study are included in the article/Supplementary Material.

## Author Contributions

TK: concept and manuscript writing. TK and JD: design, data analysis and interpretation, financial support, and manuscript editing. TK and TS: collection of the data. TK, JD, and TS: final approval of the manuscript.

## Conflict of Interest

The authors declare that the research was conducted in the absence of any commercial or financial relationships that could be construed as a potential conflict of interest.
