# Supplementary material for: High-Throughput, Temporal and Dose Dependent, Effect of Vitamins and Minerals on Chondrogenesis
Source: Front Cell Dev Biol. 2020 Feb 25;8:92. doi: 10.3389/fcell.2020.00092 (PMC7053227; doi:10.3389/fcell.2020.00092)
Supplement: Supplementary file 1 [file Data_Sheet_1.pdf]

Supplemental Data

The following table shows the presence or absence of the 14 vitamins or minerals (highlighted in yellow) which were determined to be absent in DMEM.

Supplemental Table 1 – Common media compositions

**Medium**	**DMEM**	**RPMI-1640**	**Ham's F12**	**IMDM**
**Catalog number**	D5796	R8758	51651C	I2762
**Component (g/L)**				
**Inorganic Salts**				
Calcium Chloride	0.2		0.03322	0.219
Calcium nitrate Ca(NO3)2•4H2O		0.1		
Chromium				
Cobalt (*present due to Vitamin B12)		*	*	*
Copper Sulphate $CuSO_4$ • 9H2O			0.0000025	
Ferric Nitrate • $9H_2O$	0.0001		0.000834	
Iodine				
Manganese				
Magnesium Sulfate (anhydrous)	0.09767	0.04884		0.09767
Magnesium Chloride $MgCl_2$			0.05722	
Molybdenum				
Potassium Chloride	0.4	0.4	0.224	0.33
Potassium Nitrate KNO3				0.000076
Sodium Bicarbonate NaHCO3	3.7	2	1.176	3.024
Sodium Chloride	6.4	6	7.599	4.505
Sodium Phosphate dibasic (anhydrous) Na2HPO4		0.8	0.14204	
Sodium Phosphate Monobasic (anhydrous) NaH2PO4	0.109			0.109
Sodium Selenite Na2SeO3				0.000017
Zinc ZnSO4 • 7H2O			0.000863	
**Amino Acids**				
L-Alanine			0.009	0.025
L-Arginine • HCl	0.084	0.2	0.211	0.084
L-Asparagine (Anhydrous)		0.05		0.0284
L-Asparagine • H2O			0.01501	
L-Aspartic Acid		0.02	0.0133	0.03
L-Cystine • 2HCl	0.0626	0.0652		0.09124
L-Cystine • HCl • H2O			0.035	
L-Glutamic Acid		0.02	0.0147	0.075
L-Glutamine	0.584	0.3	0.146	
Glycine	0.03	0.01	0.00751	0.03
L-Histidine (free base)		0.015		
L-Histidine • HCl • H_2O	0.042		0.02096	0.042
L-Isoleucine	0.105	0.05	0.00394	0.105
L-Leucine	0.105	0.05	0.0131	0.105
L-Lysine • HCl	0.146	0.04	0.0365	0.146
L-Methionine	0.03	0.015	0.00448	0.03
L-Phenylalanine	0.066	0.015	0.00496	0.066

L-Proline		0.02	0.0345	0.04
L-Serine	0.042	0.03	0.0105	0.042
L-Threonine	0.095	0.02	0.0119	0.095
L-Tryptophan	0.016	0.005	0.0024	0.016
L-Tyrosine • 2Na •$2H_2O$	0.10379	0.02883	0.00778	0.10379
L-Valine	0.094	0.02	0.0117	0.094
**Vitamins**				
Choline Chloride	0.004	0.003	0.01396	0.004
myo-Inositol	0.0072	0.035	0.018	0.0072
Vitamin A				
Vitamin B1 Thiamine • HCl	0.004	0.001	0.000034	0.004
Vitamin B2 Riboflavin	0.0004	0.0002	0.000038	0.0004
Vitamin B3 Niacinamide	0.004	0.001	0.0000367	0.004
Vitamin B5 D-Pantothenic Acid (hemicalcium)	0.004	0.00025	0.000238	0.004
Vitmain B6 Pyridoxal • HCl	—			0.004
Vitamin B6 Pyridoxine • HCl	0.00404	0.001	0.000062	
Vitamin B7 D-Biotin		0.0002	0.0000073	0.000013
Vitamin B9 Folic Acid	0.004	0.001	0.0013	0.004
Vitamin B12		0.000005	0.00136	0.000013
Vitamin D				
Vitamin E				
Vitamin K				
**Other**				
p-Amino Benzoic Acid		0.001		
D-Glucose	4.5	2	1.802	4.5
Glutathione (reduced)		0.001		
HEPES				5.958
Hypoxanthine sodium salt			0.00477	
Linoleic Acid			0.000084	
α-linolenic acid				
Phenol Red • Na	0.0159	0.0053	0.0013	0.016
Pyruvic Acid • Na			0.11	0.11
Putrescine •2H2O			0.000161	
Thioctic Acid			0.00021	
Thymidine			0.00073	
**Add**				
L-Glutamine				0.584

Supplemental Data 1

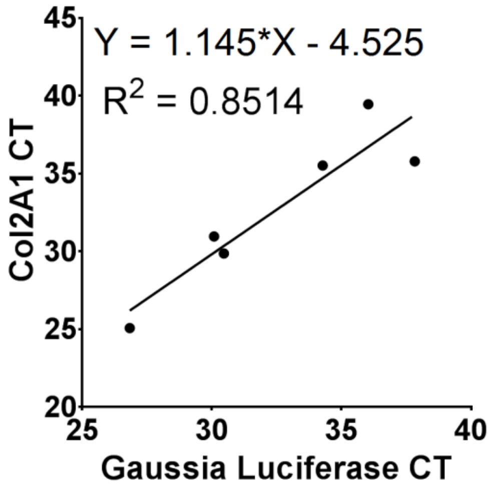

Correlation of *COL2A1* promoter-driven *Gaussia* Luciferase expression with type II collagen expression

Supplemental Data 2

The following graphs all show *COL2A1* promoter-driven expression, assessed by luminescence, at 4 time points following continuous stimulation with vitamins or minerals over a 5 log dose response (n ≥ 3 ± S.D., * > control, basal medium, * < control, basal medium; 2-way ANOVA with Sidak's multiple comparison test, Alpha 0.05). In all comparisons both day and dose were significant sources of variation with significant interaction between them (p<0.0001).

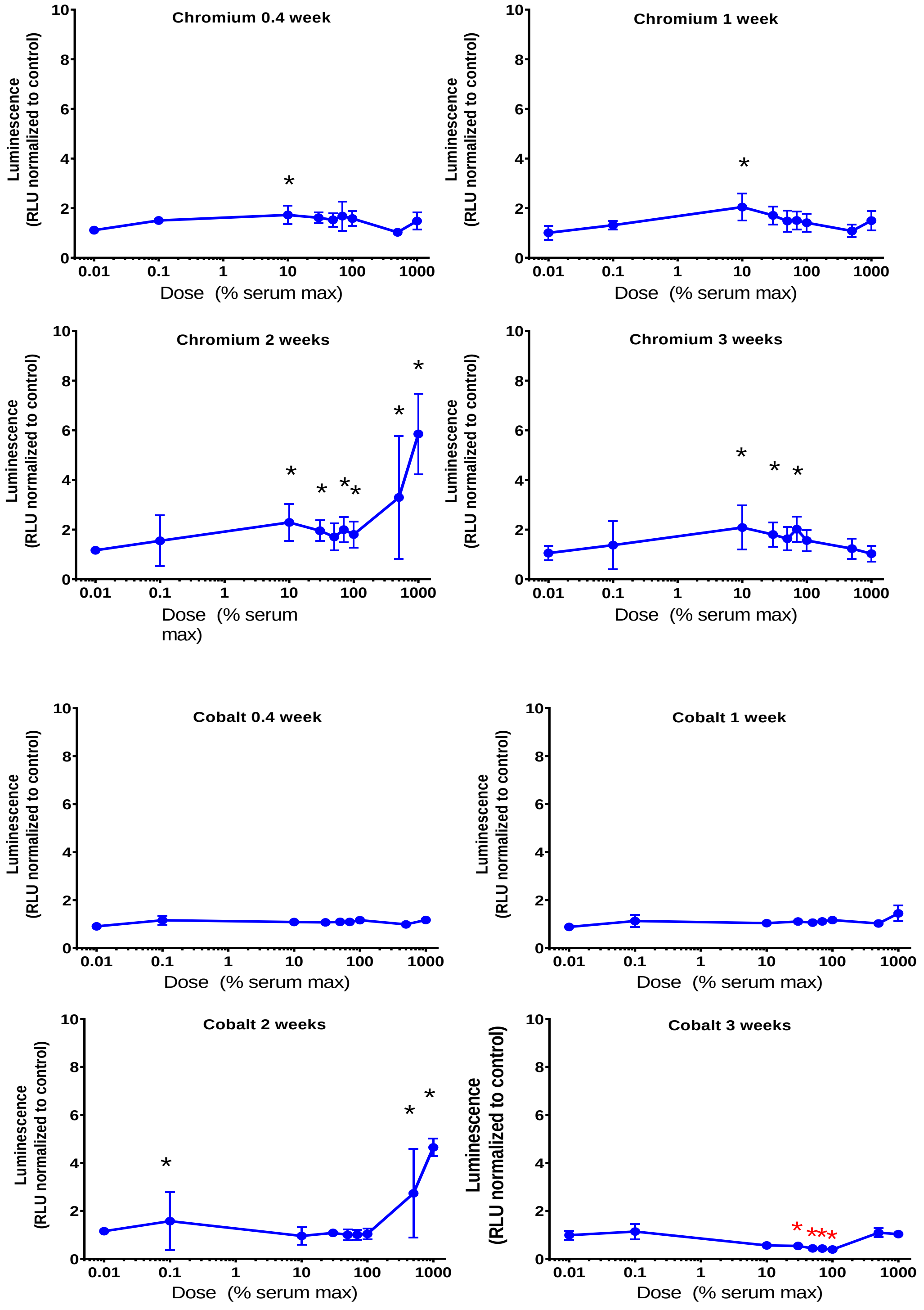

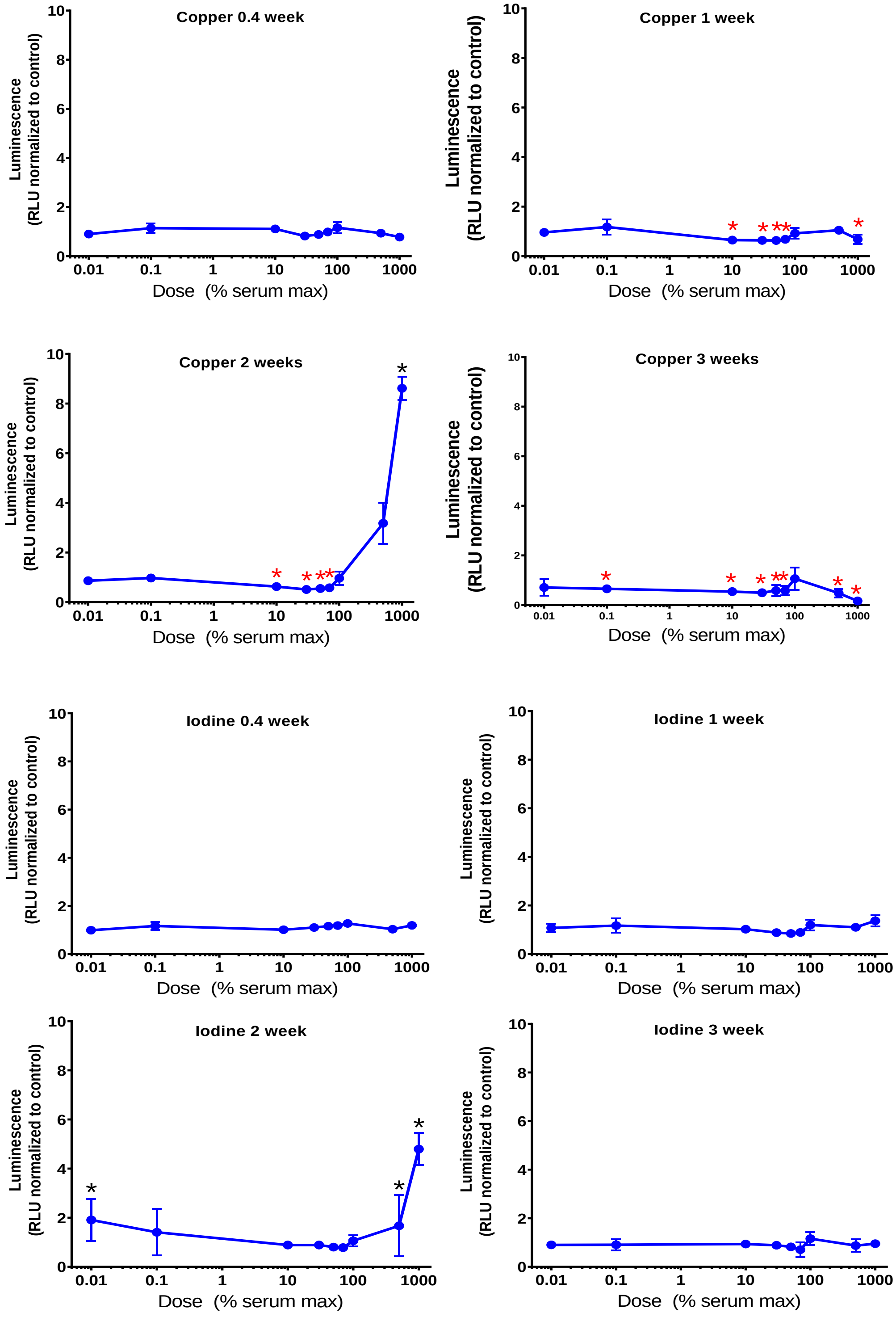
Copper 0.4 week
Copper 1 week
Copper 2 weeks
Copper 3 weeks
Iodine 0.4 week
Iodine 1 week
Iodine 2 week
Iodine 3 week
Luminescence
(RLU normalized to control)
Dose (% serum max)
0
2
4
6
8
10
0.01
0.1
1
10
100
1000
*

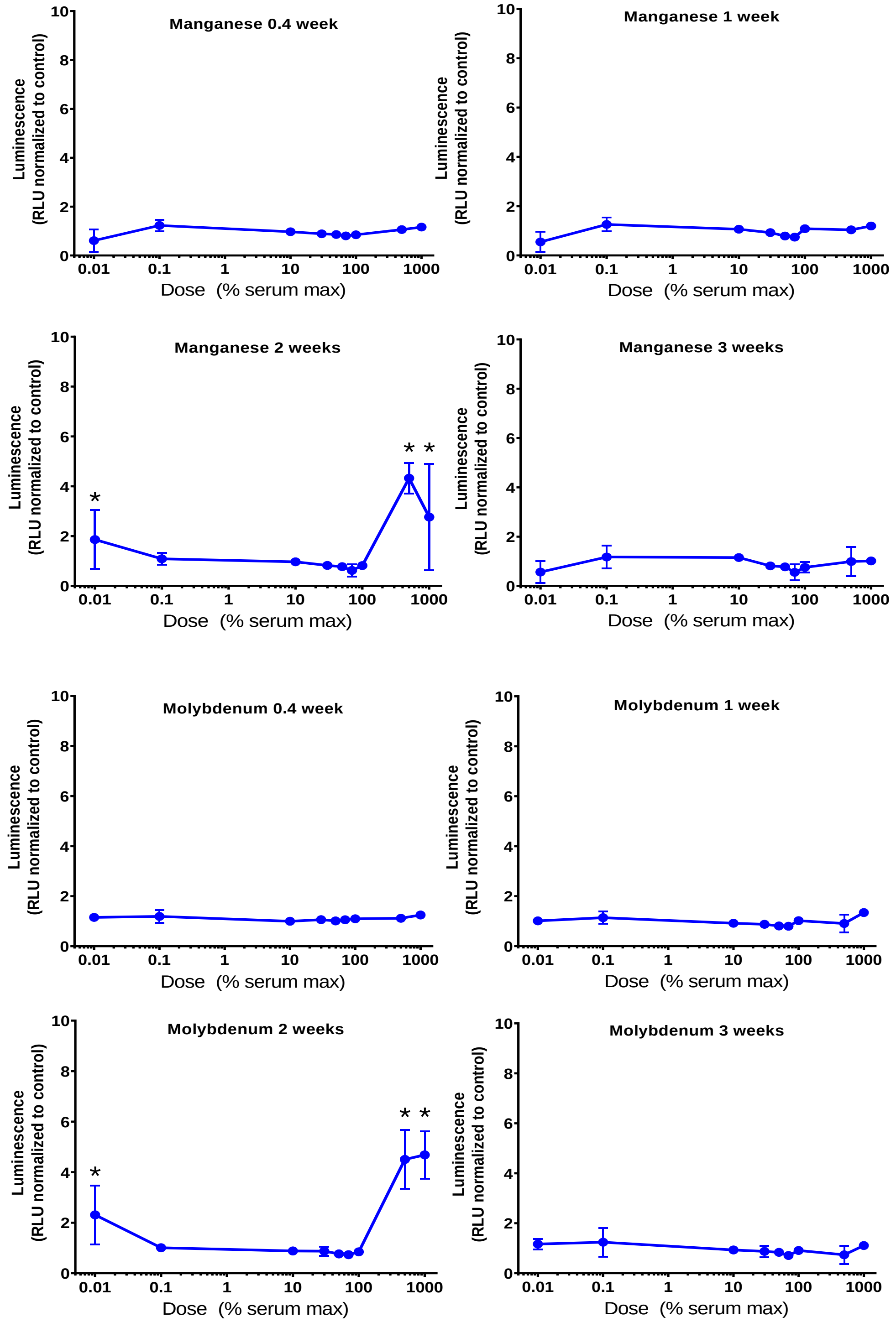
Manganese 0.4 week
Manganese 1 week
Manganese 2 weeks
Manganese 3 weeks
Molybdenum 0.4 week
Molybdenum 1 week
Molybdenum 2 weeks
Molybdenum 3 weeks
Luminescence
(RLU normalized to control)
Dose (% serum max)
0.01
0.1
1
10
100
1000
0
2
4
6
8
10
*

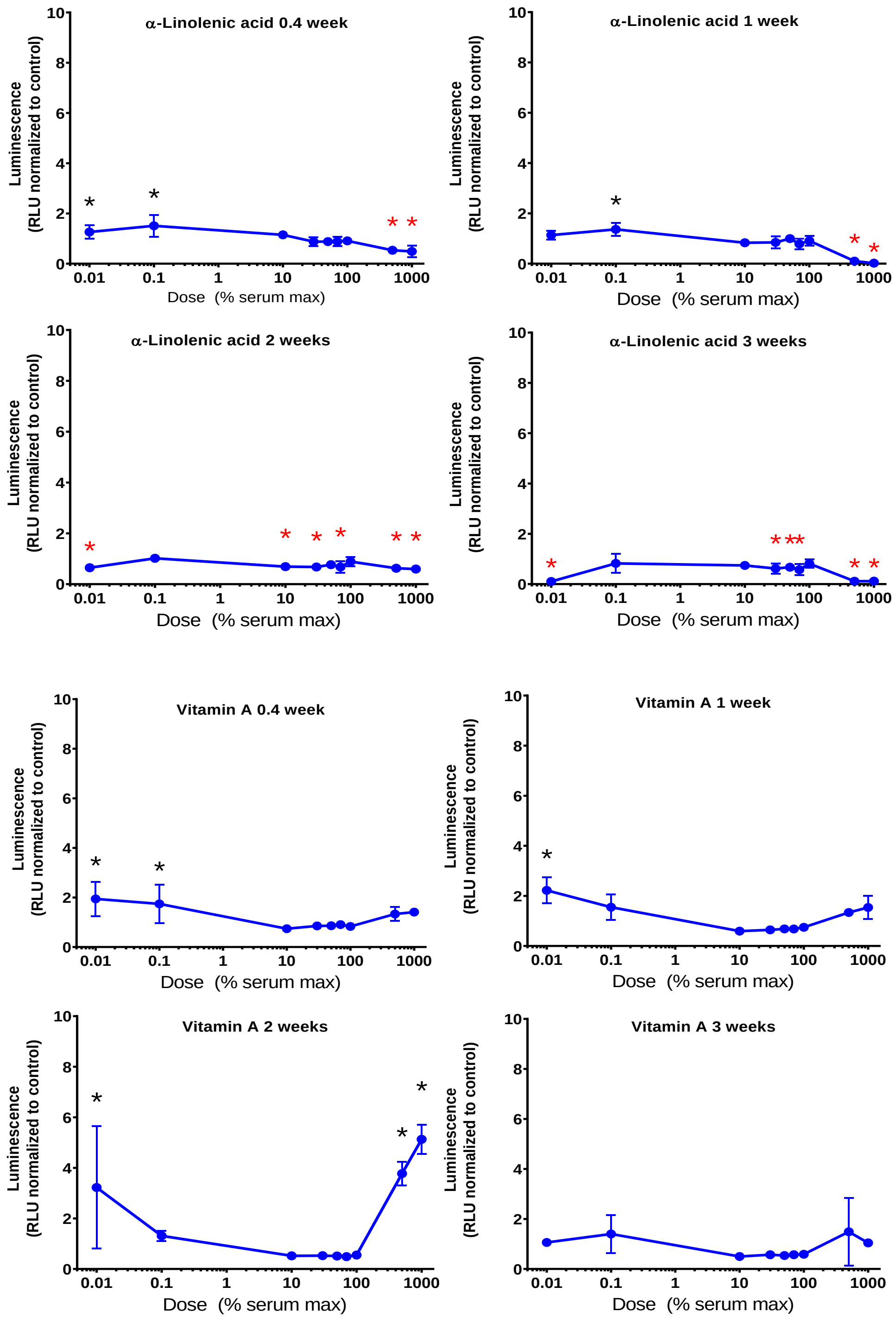
α-Linolenic acid 0.4 week
α-Linolenic acid 1 week
α-Linolenic acid 2 weeks
α-Linolenic acid 3 weeks
Vitamin A 0.4 week
Vitamin A 1 week
Vitamin A 2 weeks
Vitamin A 3 weeks
Luminescence
(RLU normalized to control)
Dose (% serum max)
0
2
4
6
8
10
0.01
0.1
1
10
100
1000
*

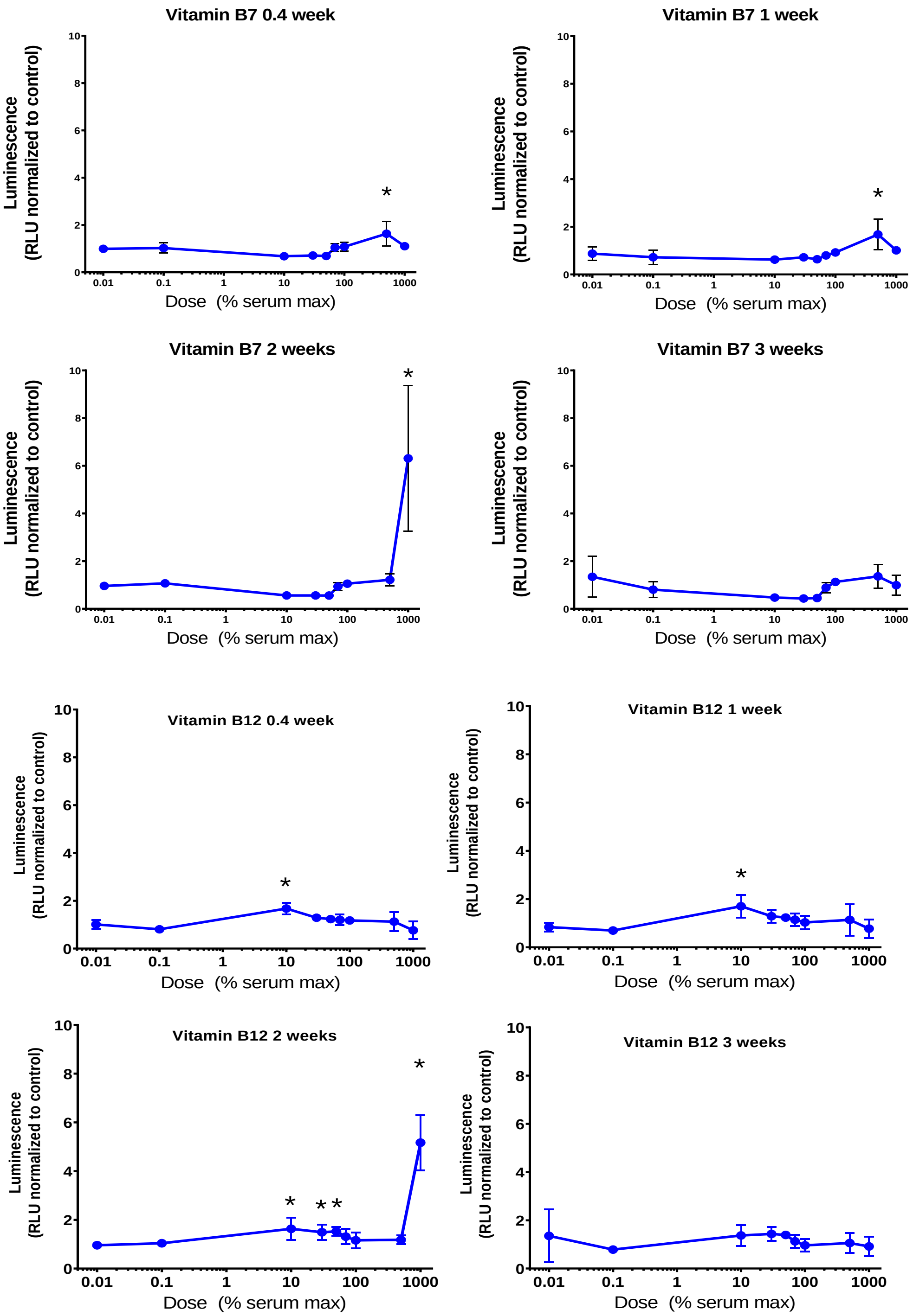
Vitamin B7 0.4 week
Vitamin B7 1 week
Vitamin B7 2 weeks
Vitamin B7 3 weeks
Vitamin B12 0.4 week
Vitamin B12 1 week
Vitamin B12 2 weeks
Vitamin B12 3 weeks
Luminescence
(RLU normalized to control)
Dose (% serum max)
0
2
4
6
8
10
0.01
0.1
1
10
100
1000
*

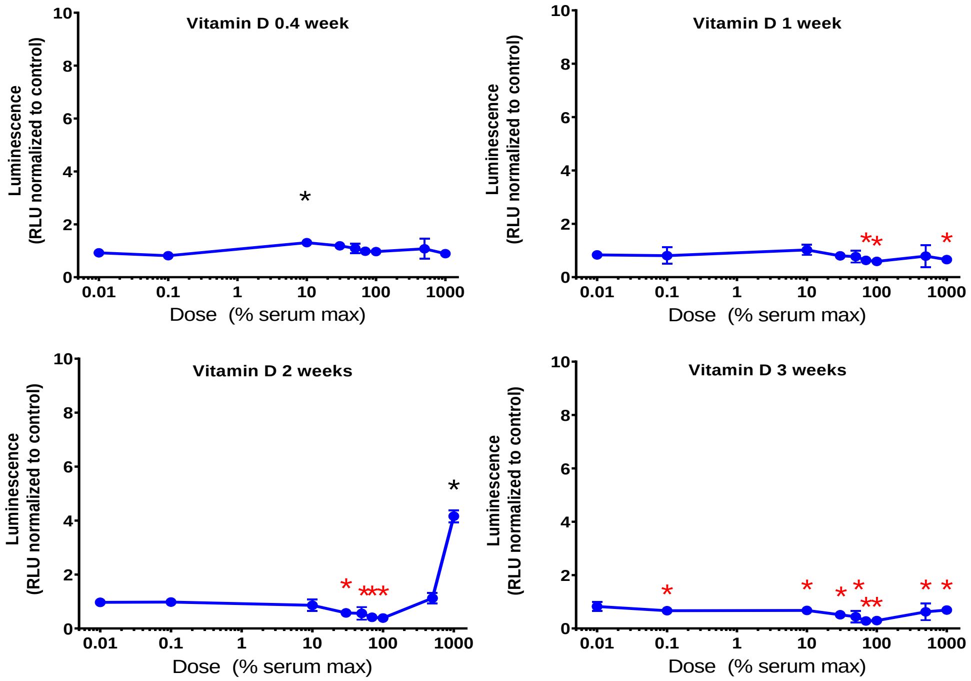

Vitamin D 0.4 week
Vitamin D 1 week
Vitamin D 2 weeks
Vitamin D 3 weeks
Luminescence
(RLU normalized to control)
Dose (% serum max)
0.01
0.1
1
10
100
1000
0
2
4
6
8
10
*

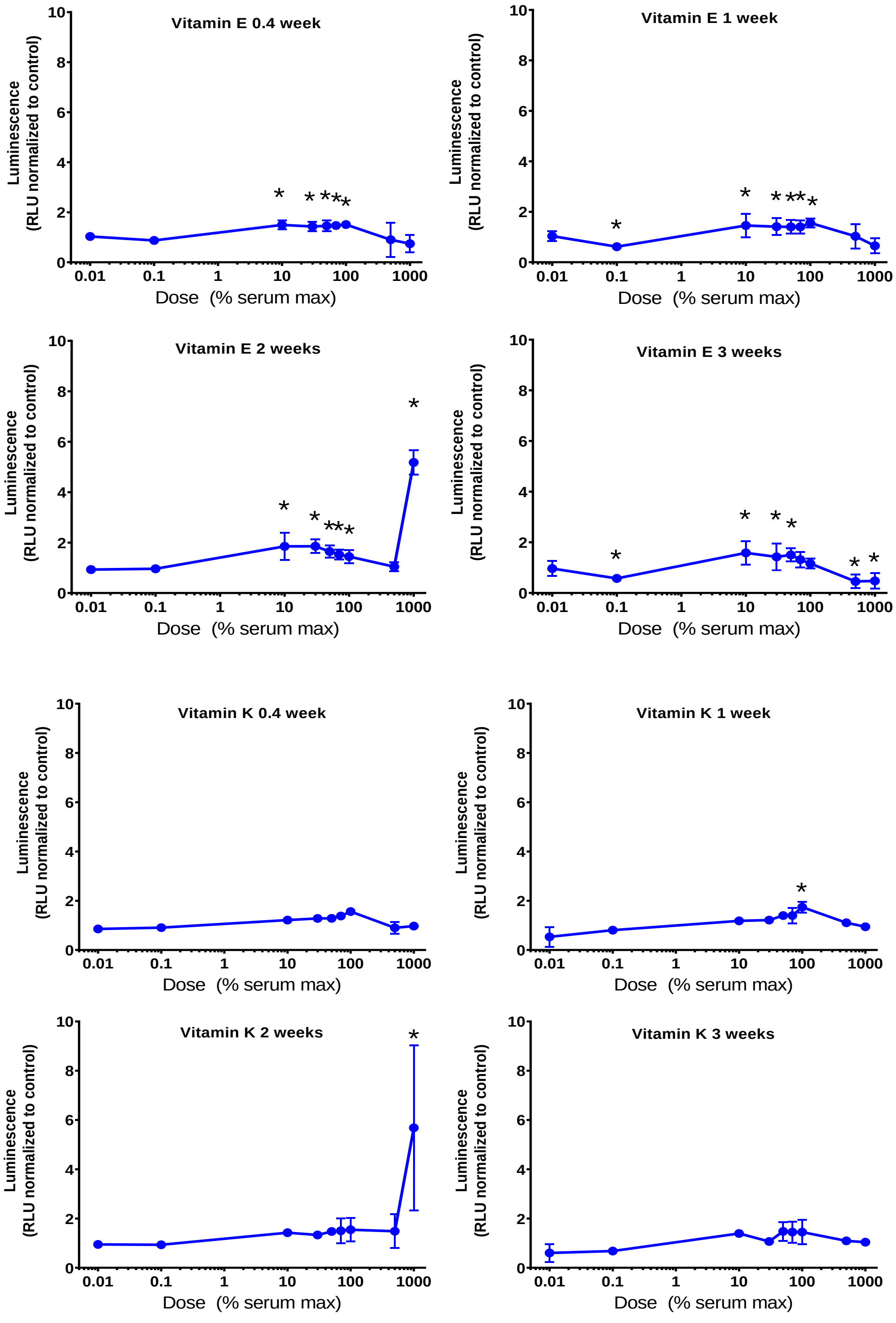
Vitamin E 0.4 week
Vitamin E 1 week
Vitamin E 2 weeks
Vitamin E 3 weeks
Vitamin K 0.4 week
Vitamin K 1 week
Vitamin K 2 weeks
Vitamin K 3 weeks
Luminescence
(RLU normalized to control)
Dose (% serum max)
0
2
4
6
8
10
0.01
0.1
1
10
100
1000
*

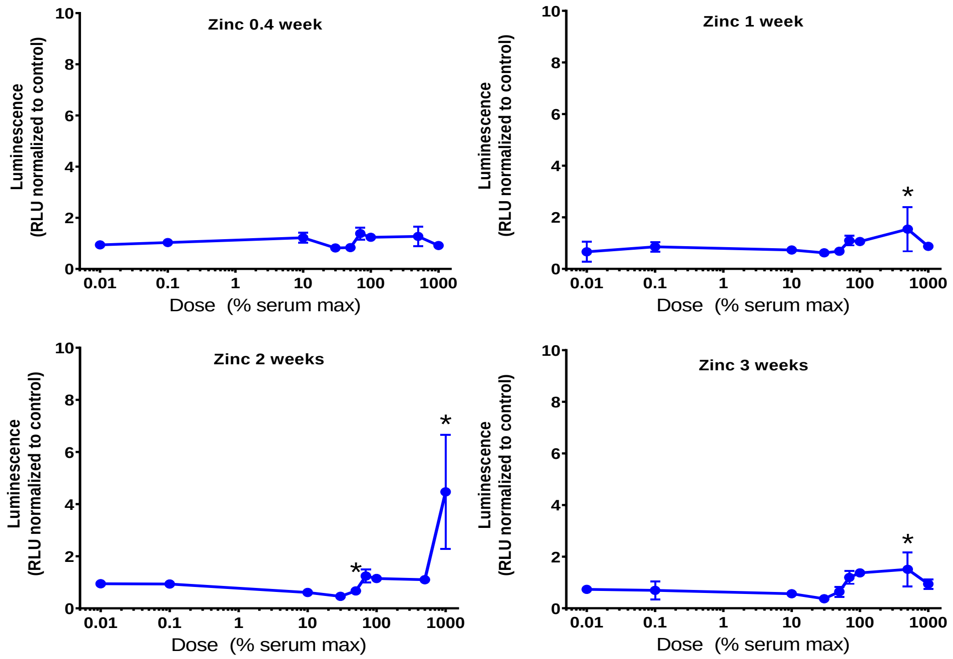
Zinc 0.4 week
Zinc 1 week
Zinc 2 weeks
Zinc 3 weeks
Luminescence
(RLU normalized to control)
Dose (% serum max)
0.01
0.1
1
10
100
1000
0
2
4
6
8
10
*

Supplemental Data 3

The following graphs show the results of supplementation of TGFβ1 containing media with vitamins and minerals alone or in combination on *COL2A1* promoter-driven. Media were normalized against basal control medium with no TGFβ1 and compared with that and the TGFβ1 supplemented medium by t-test with false discovery rate set at 1%. TGFβ1 supplemented medium is represented by a red dashed line. Conditions that were statistically greater than TGFβ1 supplemented medium are represented by a ▲ and an *; conditions that are greater than basal but not TGFβ1 are represented by ●; conditions that are greater than basal but lower than TGFβ1 are represented by ▼ and an L; conditions that were not significantly different to basal are represented by ■ (these are also significantly lower than TGFβ1 after 0.4 week); there were no conditions that were significantly lower than basal medium alone.

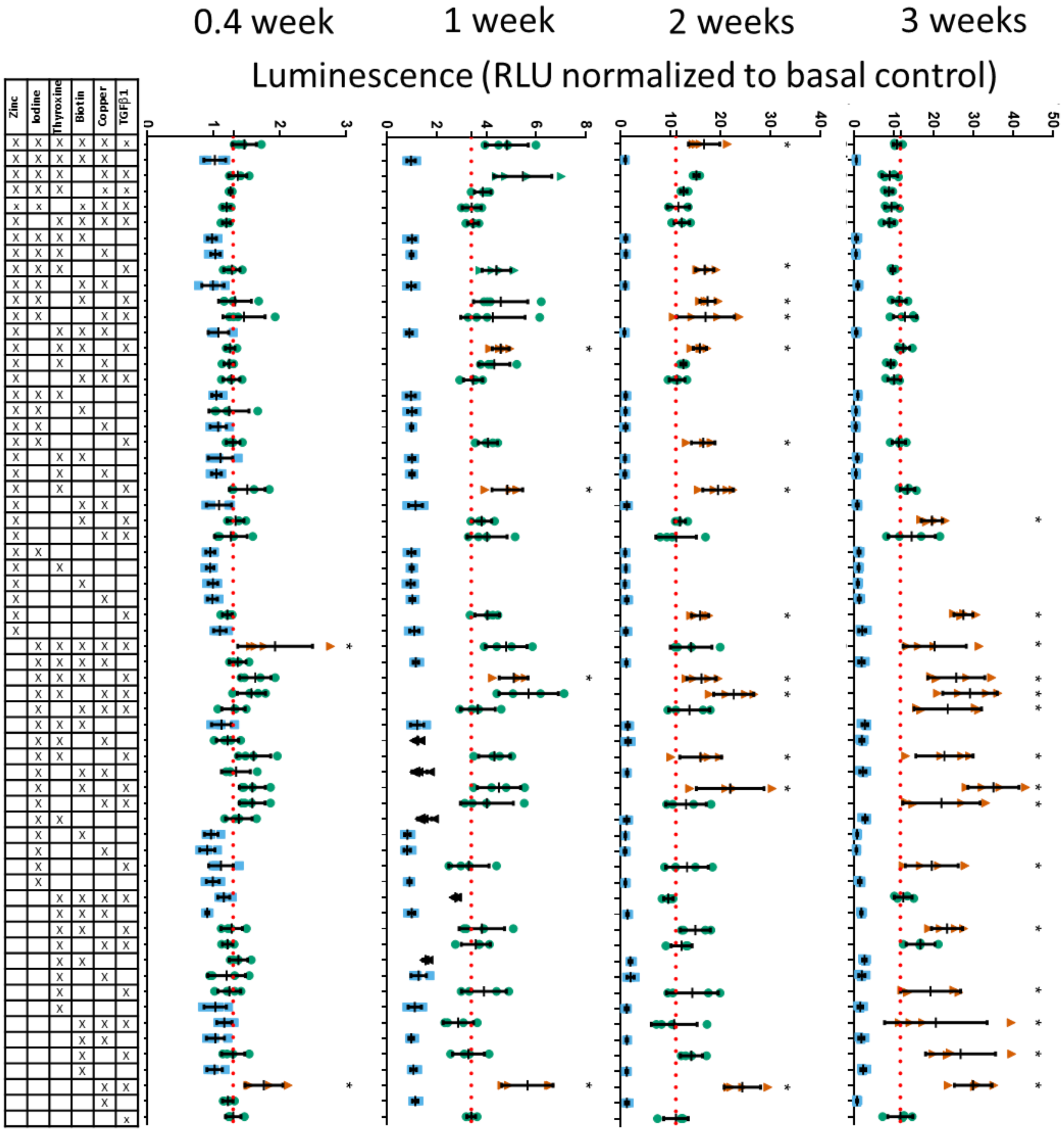

Supplemental Data 4

The following graphs show the results of addition of vitamins B12, E, and chromium to previously defined supplemented media over a three-week period on *COL2A1* promoter-driven expression. Media were normalized against basal control medium with no TGFβ1 and compared to each supplemented media by two-way ANOVA with Sidak's multiple comparison test, Alpha 0.05. Each supplemented media average is represented by a red dashed line. Conditions that were statistically greater than their respective supplemented medium are represented by a ▲ and an *; conditions that were similar to their respective supplemented medium are represented by a ●; conditions that were lower than their respective supplemented medium are represented by ▼. There was significant interaction between the supplemented media and the additional factors, both of which were also significant sources of variation.

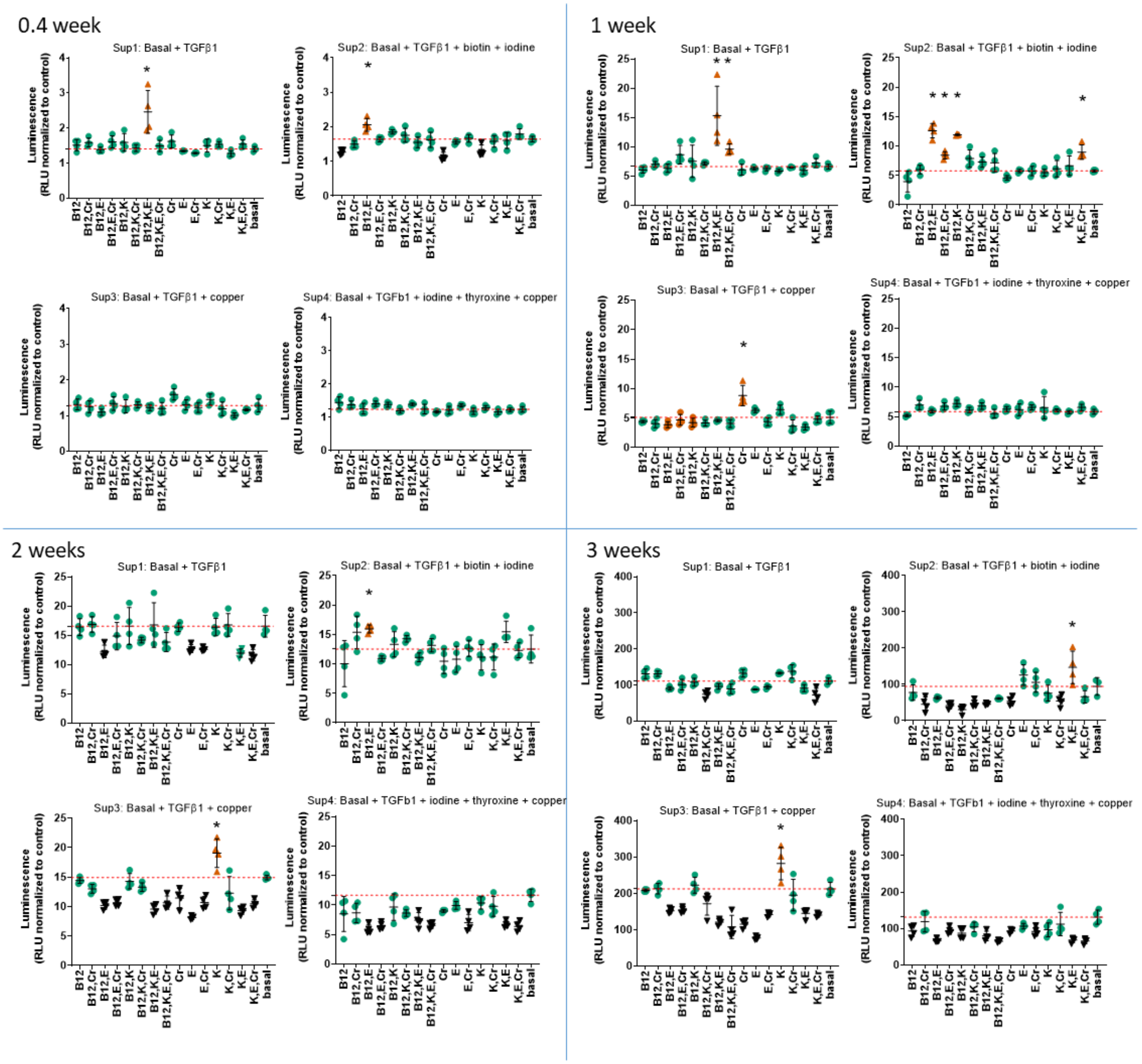

Supplemental Data 5

The following graphs all show *COL2A1* promoter-driven expression, assessed by luminescence, at 4 time points following continuous stimulation with vitamins or minerals in the presence of TGFβ1 (1ng/ml) over a 5 log dose response. Conditions that were statistically greater than TGFβ1 supplemented medium are represented by a ▲ and an *; conditions that were similar to TGFβ1 supplemented medium are represented by a ●; conditions that were lower than TGFβ1 supplemented medium are represented by ▼; 2-way ANOVA with Sidak's multiple comparison test, Alpha 0.05. A red dashed line indicates the mean TGFβ1 supplemented medium response. In most comparisons both time and dose were significant sources of variation with significant interaction between them, where this was not the case details are noted below the set of graphs.

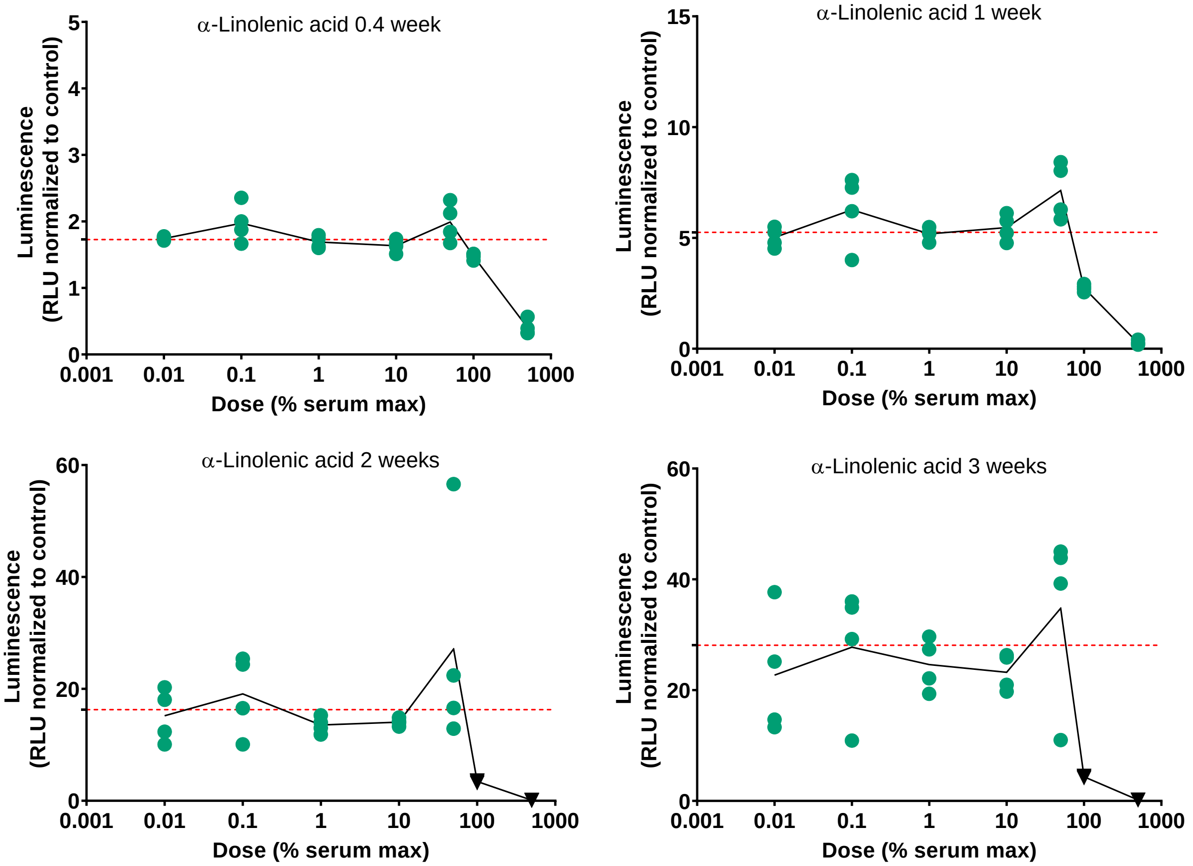

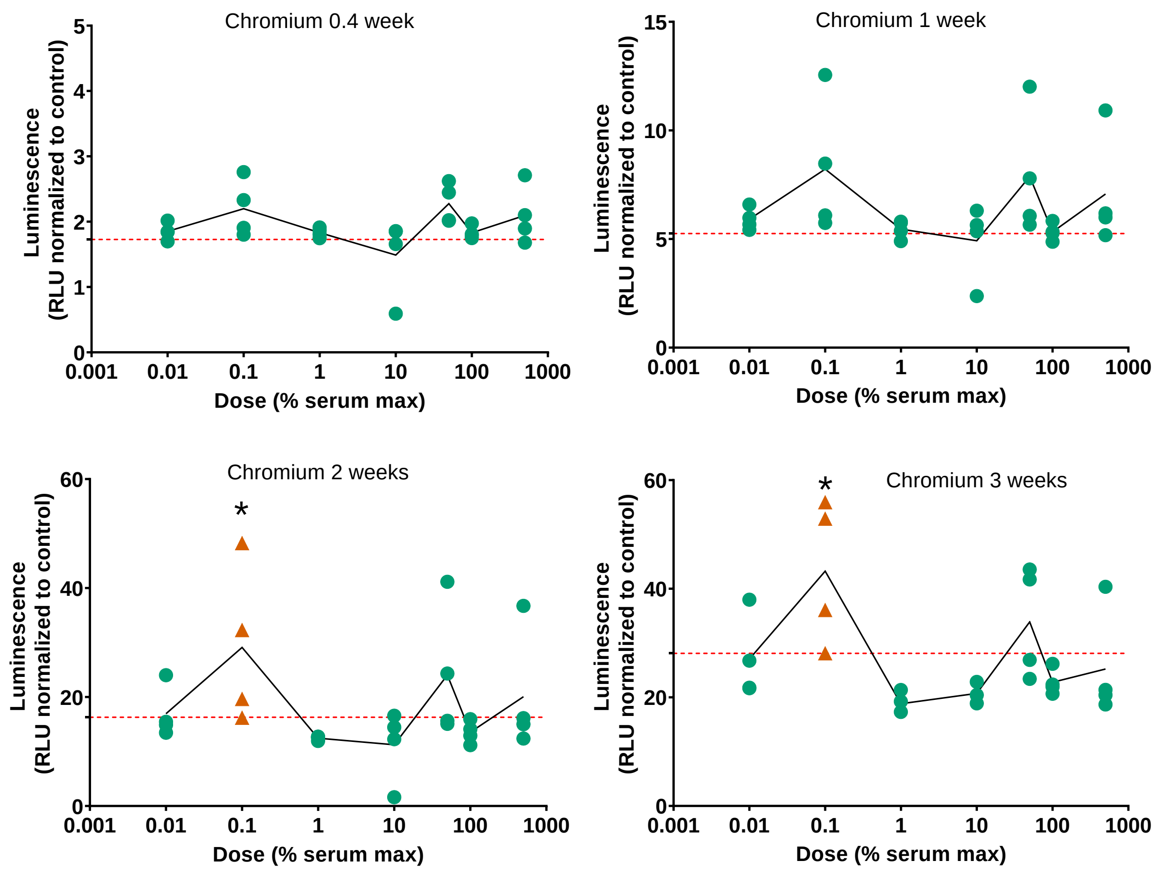
Chromium 0.4 week
Chromium 1 week
Chromium 2 weeks
Chromium 3 weeks
Luminescence
(RLU normalized to control)
Dose (% serum max)
0.001
0.01
0.1
1
10
100
1000
*

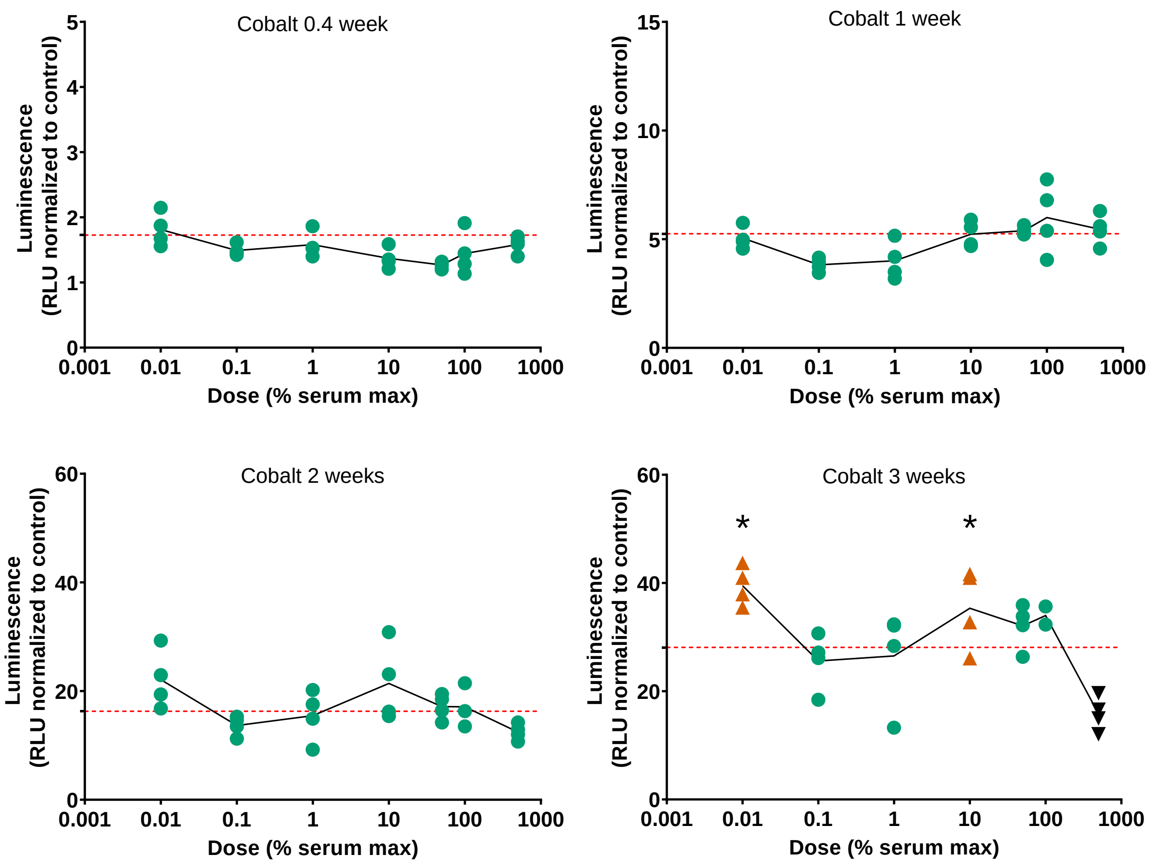

Cobalt 0.4 week
Cobalt 1 week
Cobalt 2 weeks
Cobalt 3 weeks
Luminescence
(RLU normalized to control)
Dose (% serum max)
0.001
0.01
0.1
1
10
100
1000
*

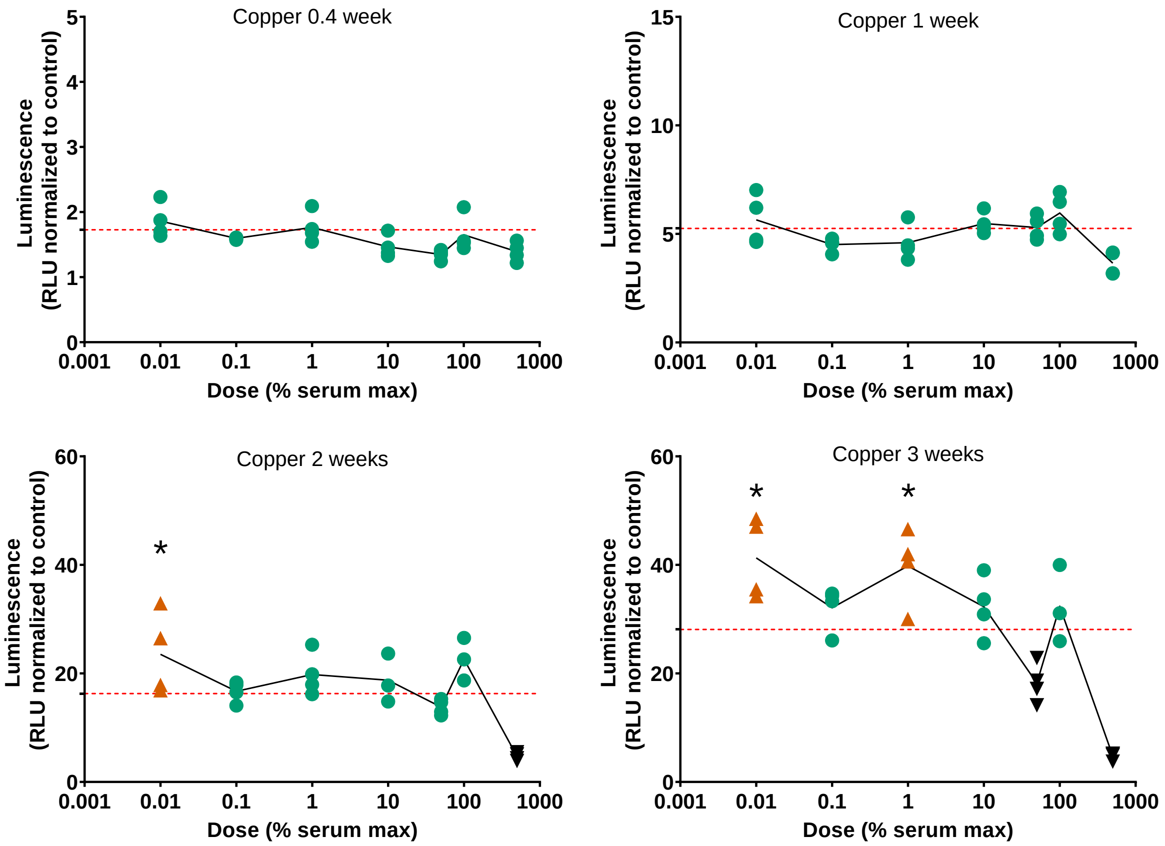

Copper 0.4 week
Copper 1 week
Copper 2 weeks
Copper 3 weeks
Luminescence
(RLU normalized to control)
Dose (% serum max)
0.001
0.01
0.1
1
10
100
1000
*

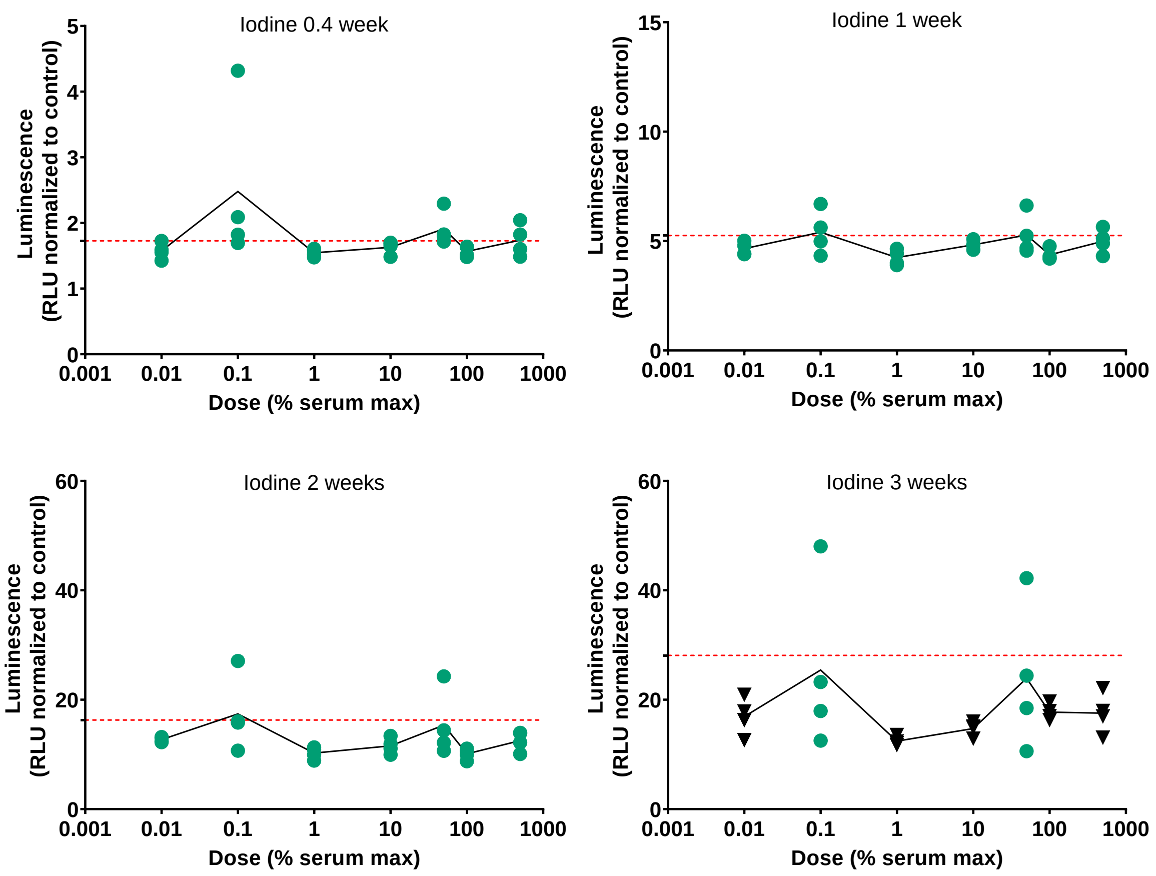

There was no significant interaction found between time and dose, both time ($p < 0.0001$) and dose ($p = 0.0007$) were significant sources of variation.

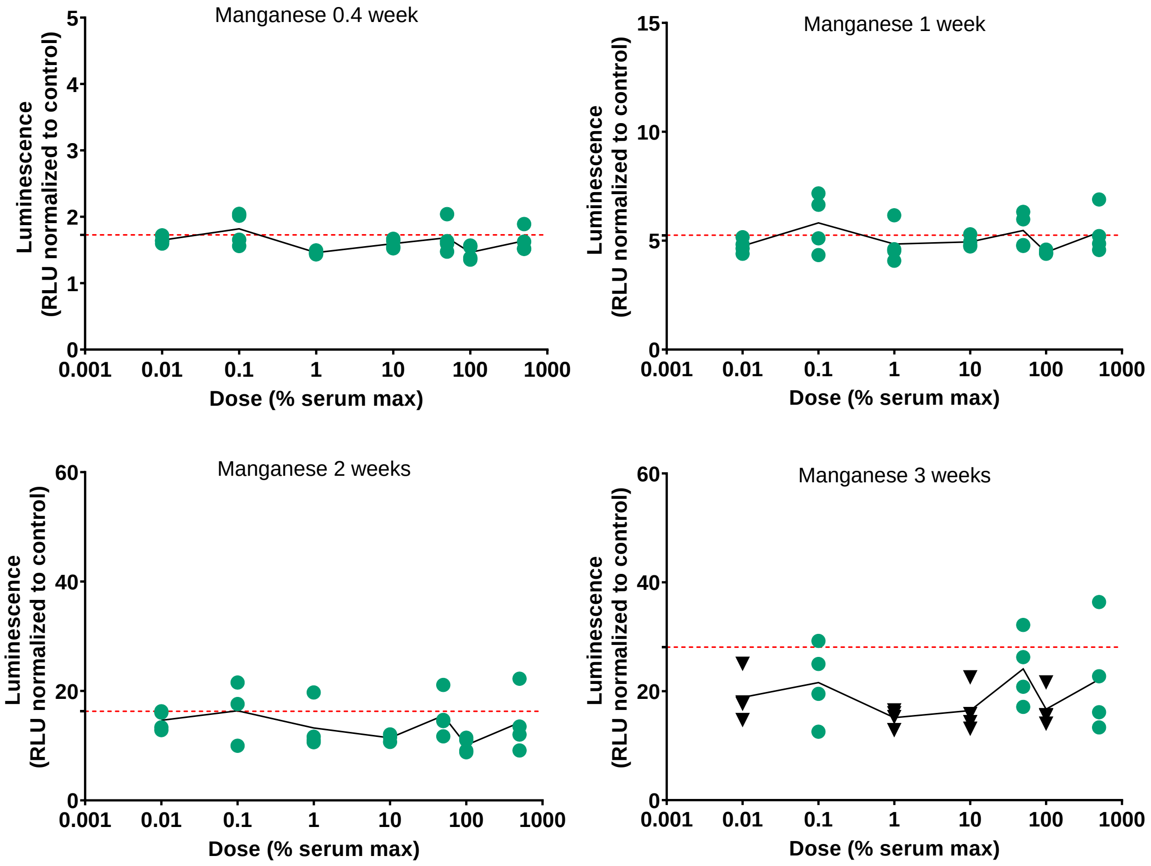

There was no significant interaction found between time and dose; both time ($p < 0.0001$) and dose ($p = 0.0024$) were significant sources of variation.

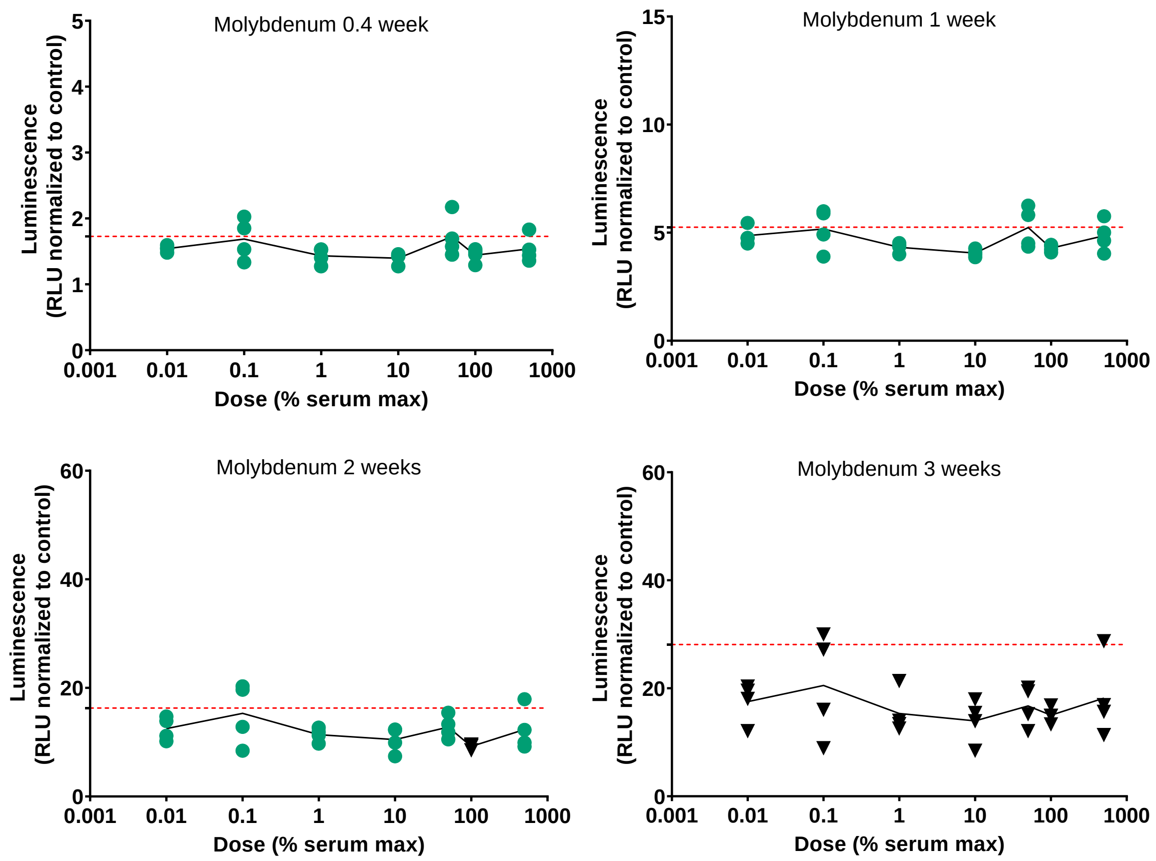
Molybdenum 0.4 week
Luminescence (RLU normalized to control)
Dose (% serum max)
Molybdenum 1 week
Luminescence (RLU normalized to control)
Dose (% serum max)
Molybdenum 2 weeks
Luminescence (RLU normalized to control)
Dose (% serum max)
Molybdenum 3 weeks
Luminescence (RLU normalized to control)
Dose (% serum max)

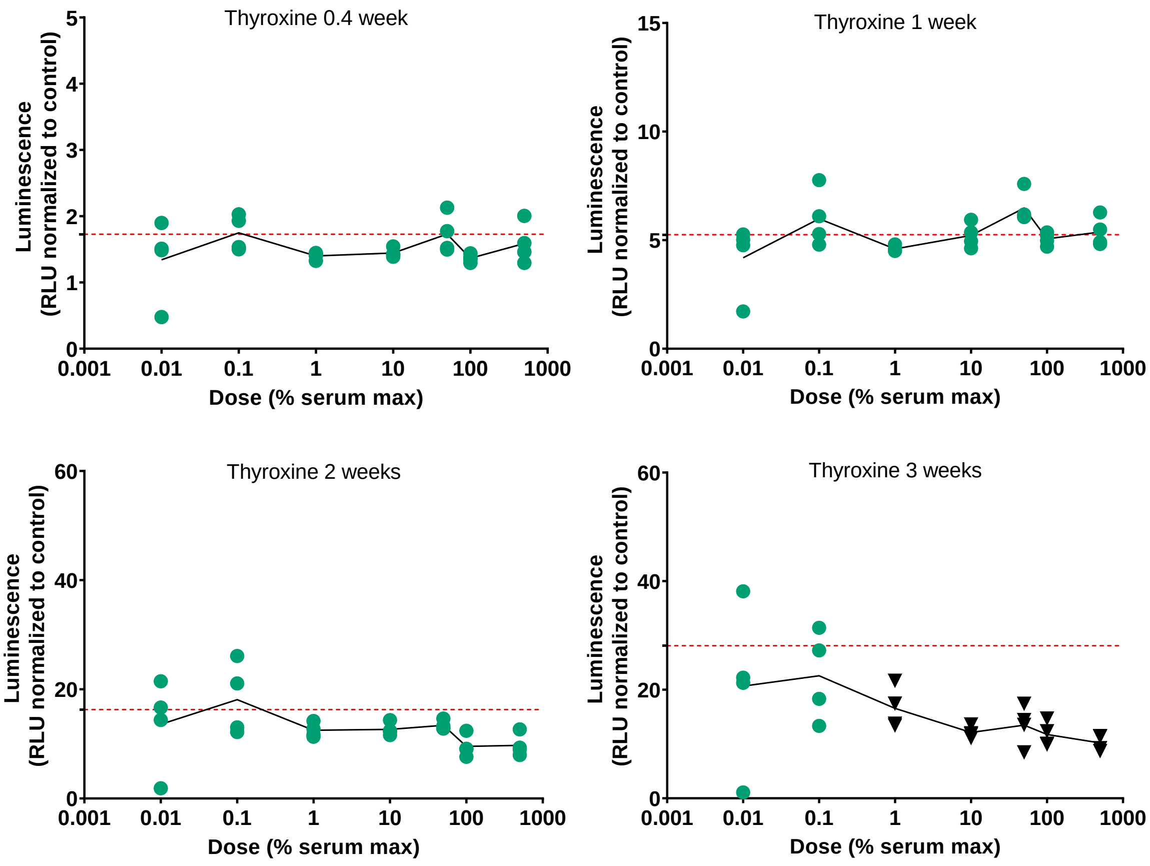
Thyroxine 0.4 week
Thyroxine 1 week
Thyroxine 2 weeks
Thyroxine 3 weeks
Luminescence
(RLU normalized to control)
Dose (% serum max)
0.001
0.01
0.1
1
10
100
1000
0
1
2
3
4
5
10
15
20
40
60

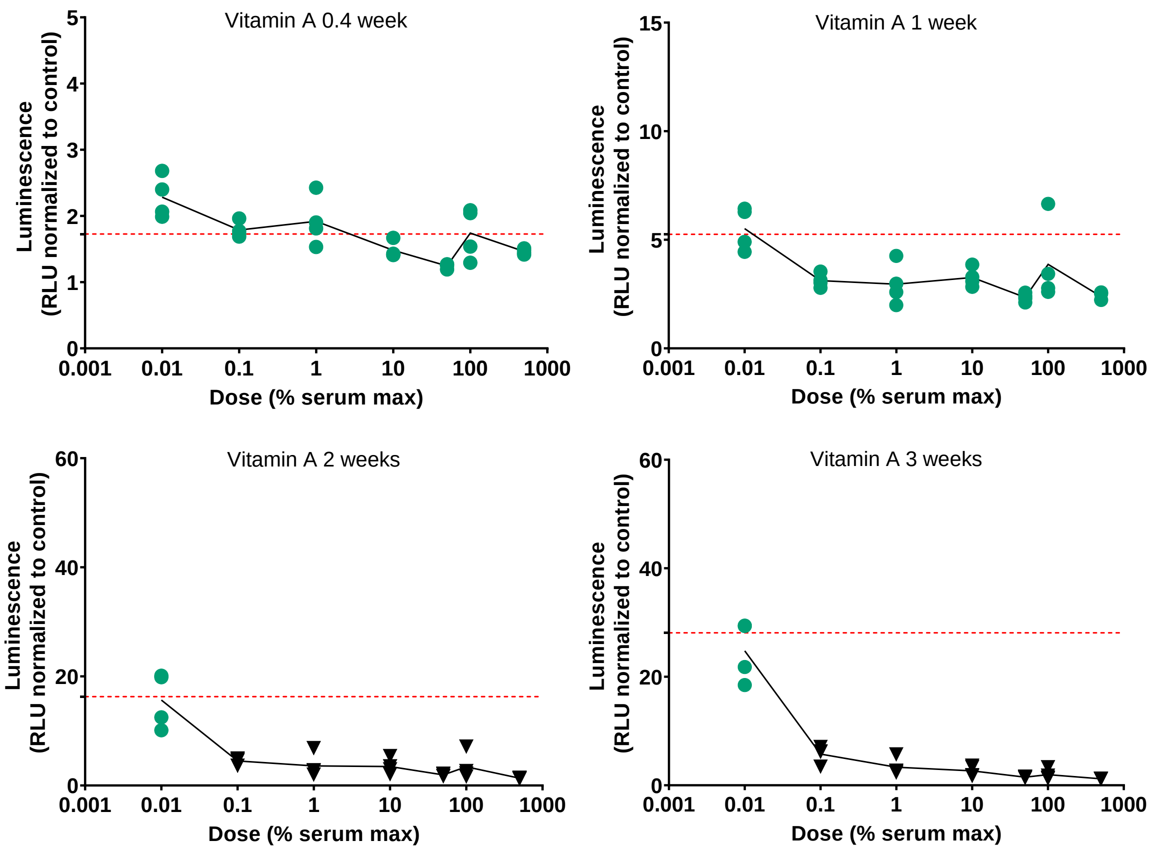
Vitamin A 0.4 week
Vitamin A 1 week
Vitamin A 2 weeks
Vitamin A 3 weeks
Luminescence
(RLU normalized to control)
Dose (% serum max)
0.001
0.01
0.1
1
10
100
1000

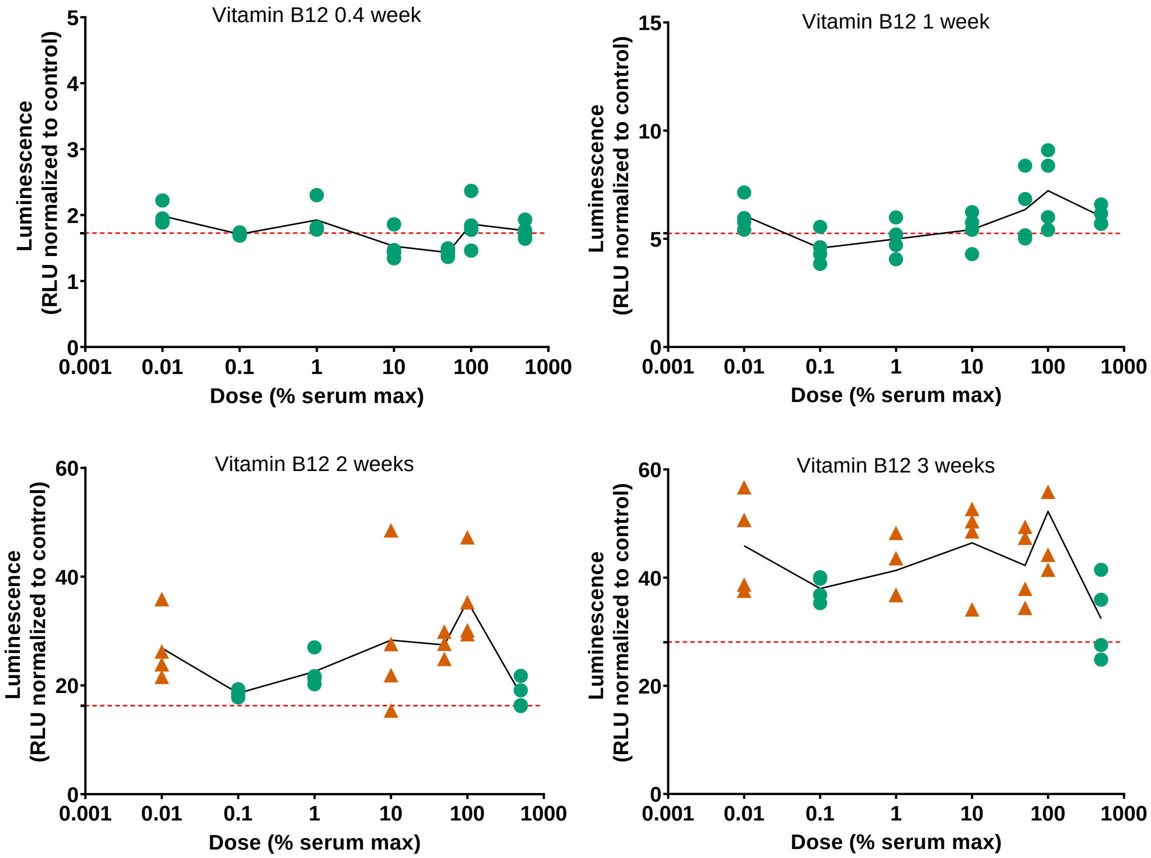
Vitamin B12 0.4 week
Vitamin B12 1 week
Vitamin B12 2 weeks
Vitamin B12 3 weeks
Luminescence
(RLU normalized to control)
Dose (% serum max)
0.001
0.01
0.1
1
10
100
1000
0
1
2
3
4
5
10
15
20
40
60

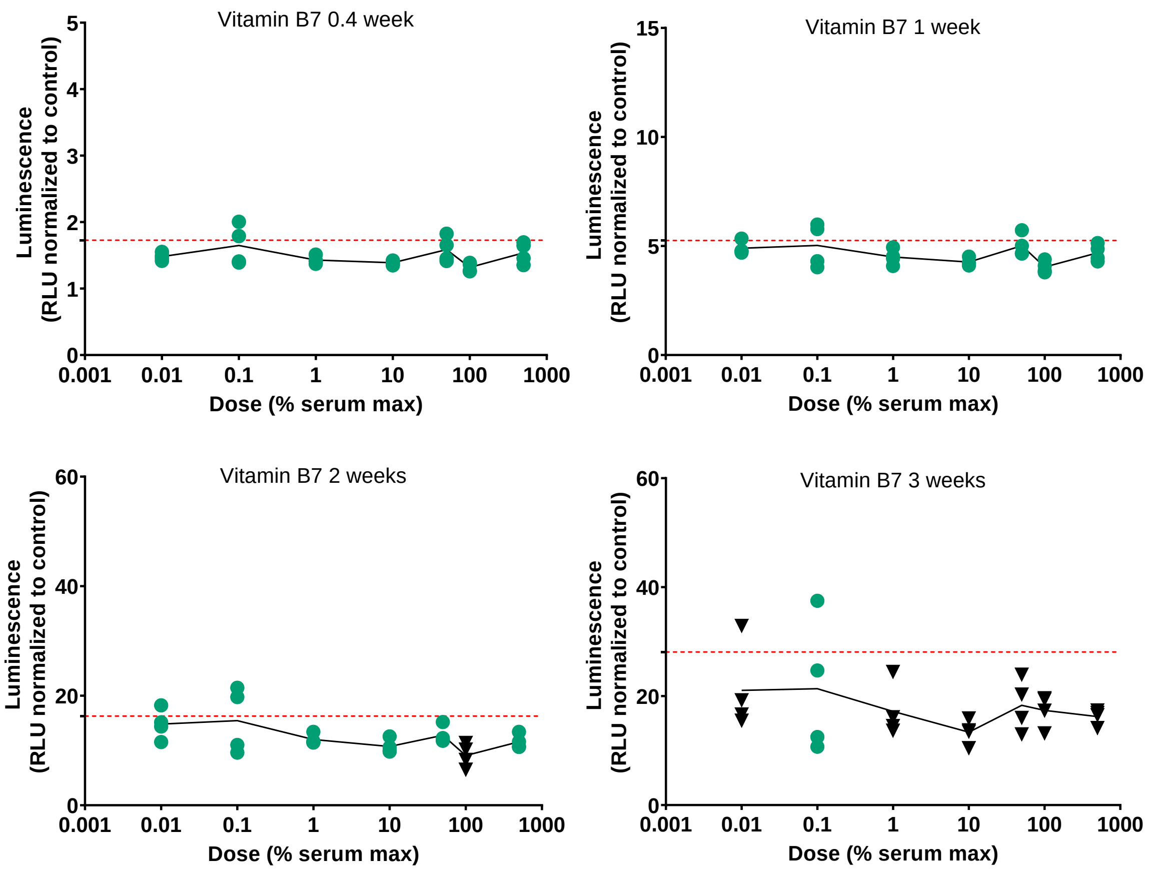

There was no significant interaction found between time and dose; both time ($p < 0.0001$) and dose ($p = 0.0005$) were significant sources of variation.

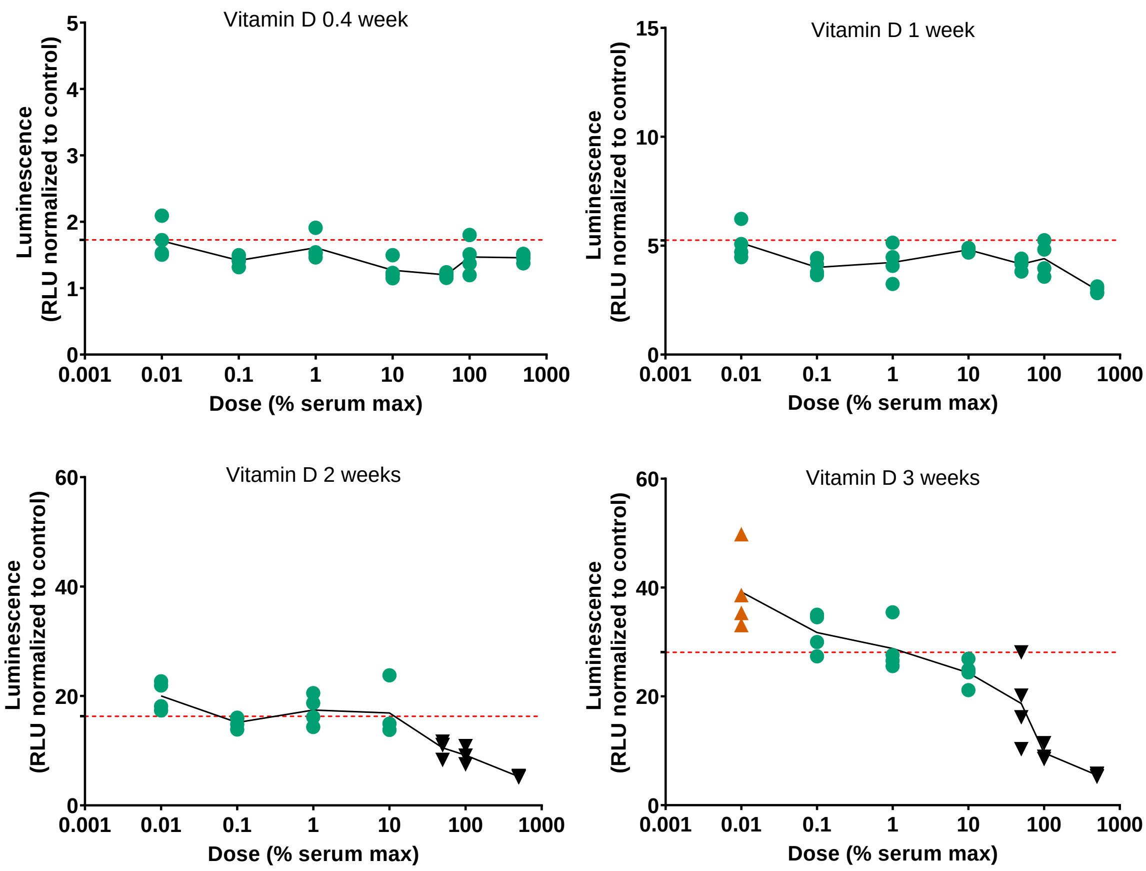

Vitamin D 0.4 week
Vitamin D 1 week
Vitamin D 2 weeks
Vitamin D 3 weeks
Luminescence (RLU normalized to control)
Dose (% serum max)
0.001
0.01
0.1
1
10
100
1000

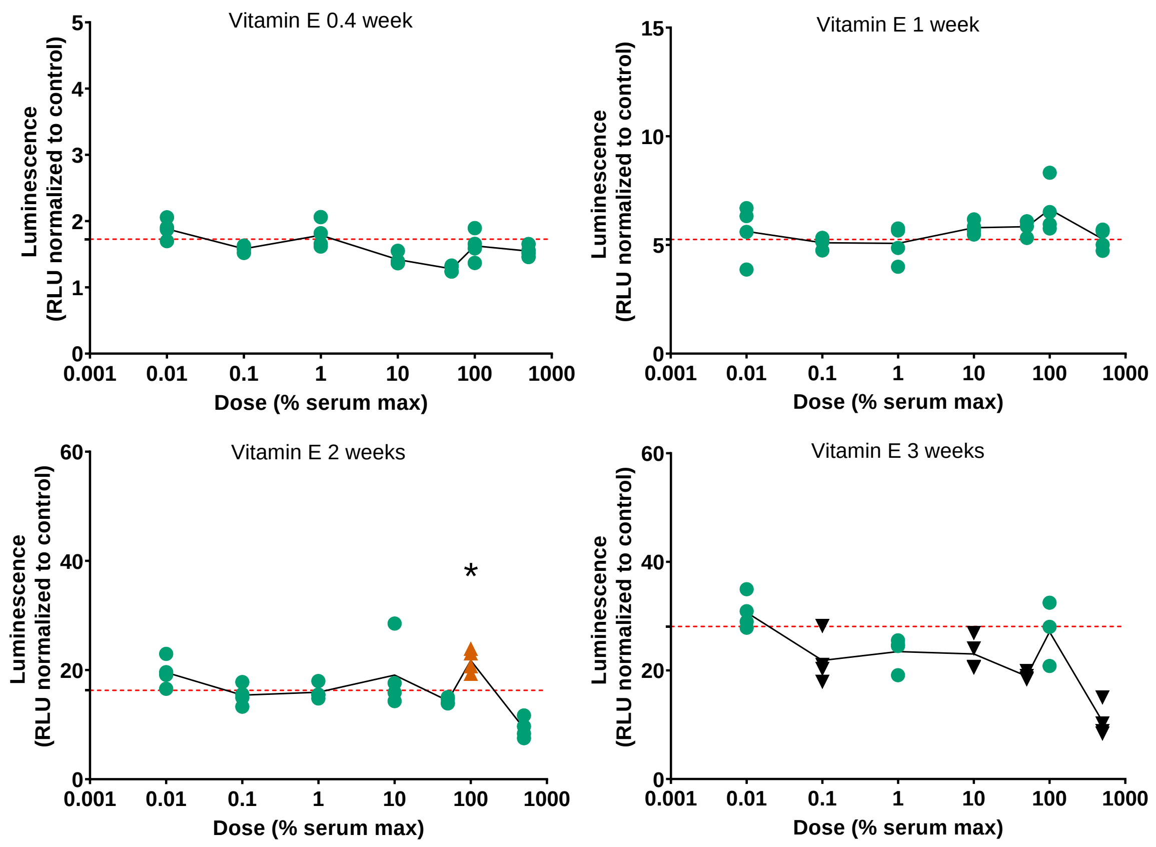

Vitamin E 0.4 week
Vitamin E 1 week
Vitamin E 2 weeks
Vitamin E 3 weeks
Luminescence
(RLU normalized to control)
Dose (% serum max)
0.001
0.01
0.1
1
10
100
1000
*

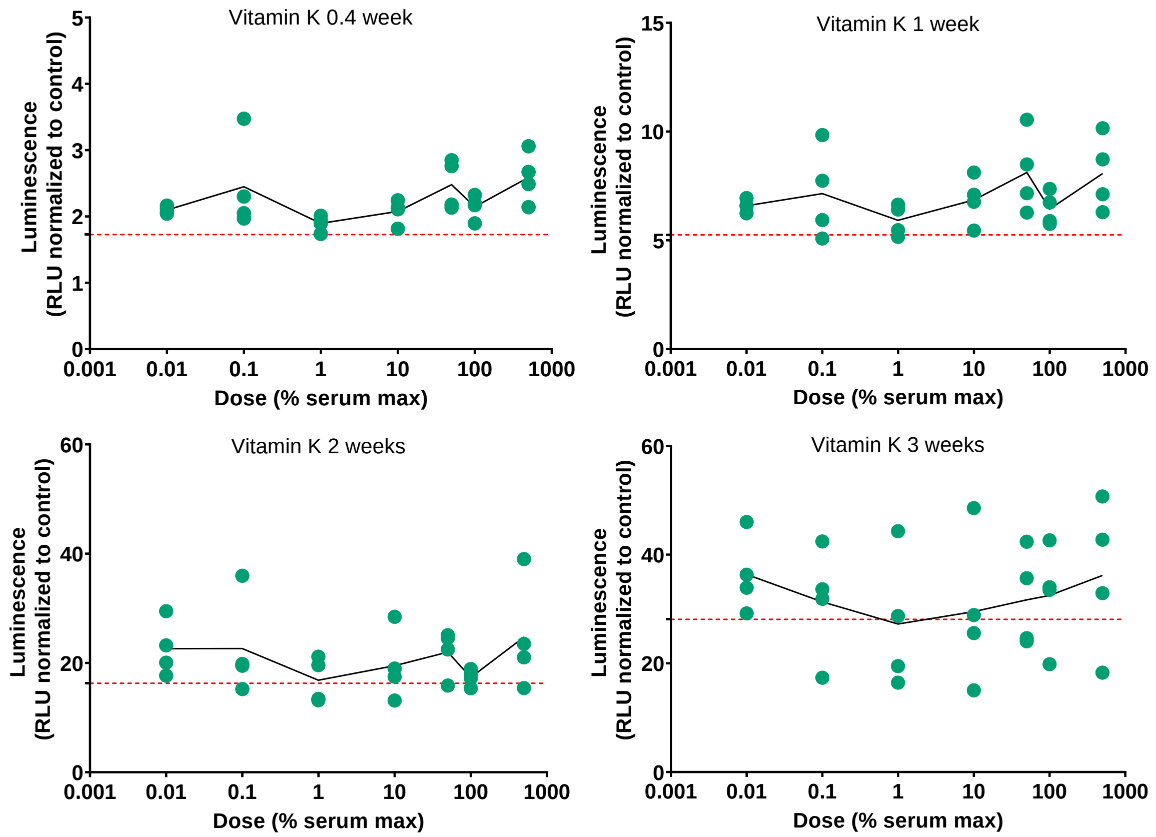

There was no significant interaction found between time and dose; only time ($p < 0.0001$) was a significant source of variation.

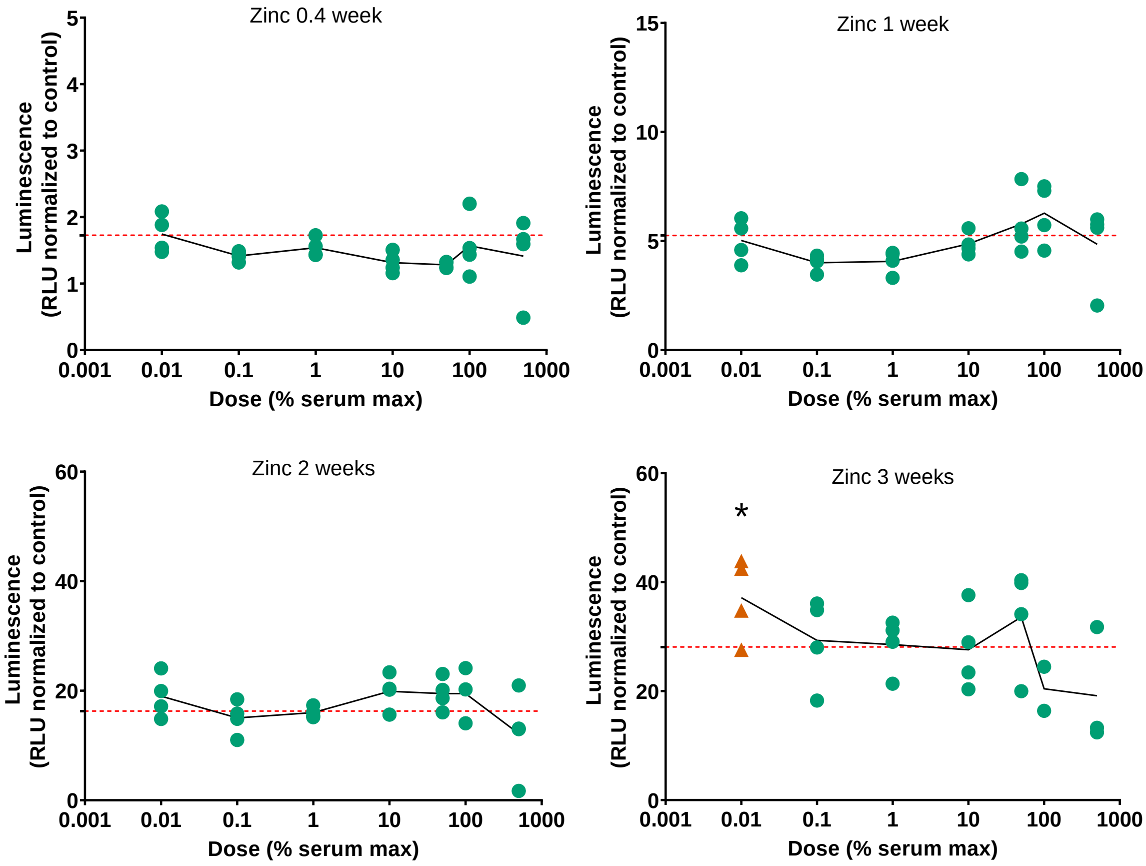

Zinc 0.4 week
Zinc 1 week
Zinc 2 weeks
Zinc 3 weeks
*
Luminescence
(RLU normalized to control)
Dose (% serum max)
0.001
0.01
0.1
1
10
100
1000

Supplemental Data 6

The following graphs all show metabolic activity, assessed by resazurin fluorescence, at day 21 following continuous stimulation with vitamins or minerals in the presence of TGFβ1 (1ng/ml) over a 5 log dose response. Conditions that were statistically greater than TGFβ1 supplemented medium are represented by a ▲ and an *; conditions that were similar to TGFβ1 supplemented medium are represented by a ●; conditions that were lower than TGFβ1 supplemented medium are represented by ▼; 1-way ANOVA with Dunnett's multiple comparison test, Alpha 0.05. A red dashed line indicates the mean TGFβ1 supplemented medium response.

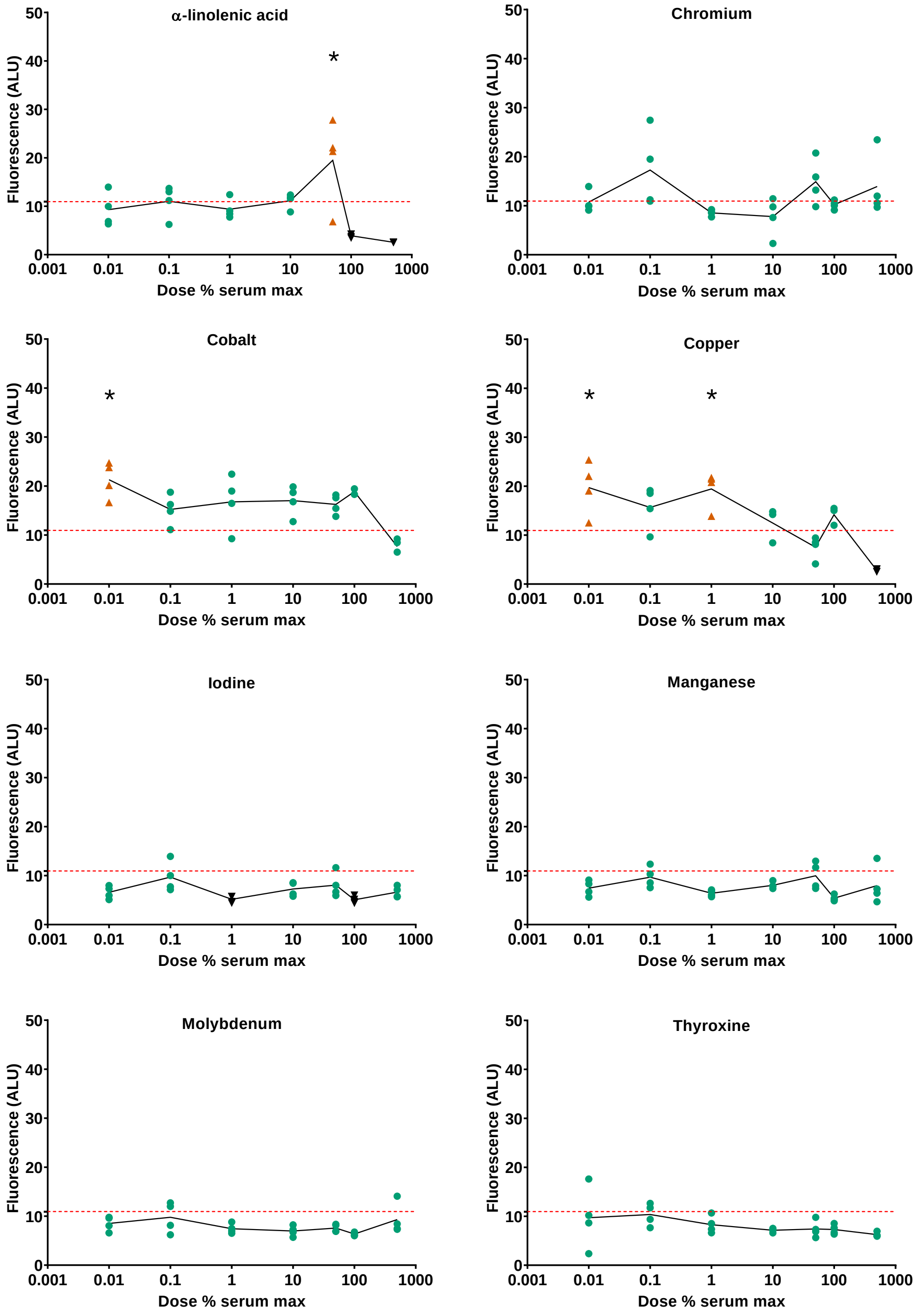

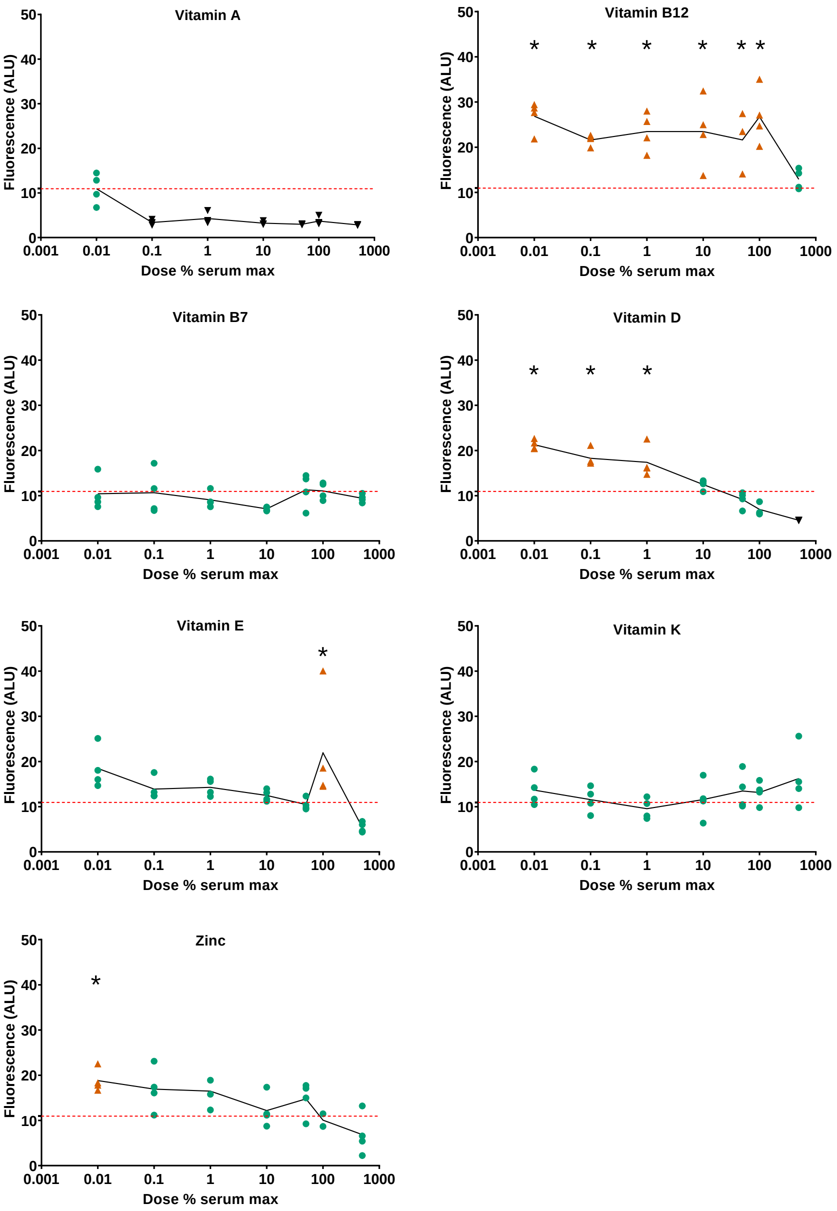
Vitamin A
Vitamin B12
Vitamin B7
Vitamin D
Vitamin E
Vitamin K
Zinc
Fluorescence (ALU)
Dose % serum max
0
10
20
30
40
50
0.001
0.01
0.1
1
10
100
1000
*

Supplemental Data 7

The following table shows the cumulative response (normalized to basal medium control) to vitamin and mineral supplementation over the 21-day duration of the experiment in the presence of TGFβ1 (1ng/ml), the table is sorted in order of the highest net area.

Factor (dose % serum max)	Net Area	Std. Error	95% Confidence Interval
Vitamin B12 (100)	434.3	55.07	326.4 to 542.3
Chromium (0.1)	369.1	84.07	204.3 to 533.9
Vitamin B12 (10)	355.2	76.11	206.0 to 504.4
Vitamin B12 (0.01)	348.2	42.19	265.5 to 430.9
Vitamin B12 (50)	341.9	26.09	290.8 to 393.0
α-linolenic acid (50)	323	111.3	104.9 to 541.2
Vitamin K (500)	317.3	66.09	187.8 to 446.8
Copper (0.01)	308.1	44.43	221.0 to 395.2
Chromium (50)	303.8	69.33	167.9 to 439.7
Vitamin B12 (1)	297.7	22.98	252.6 to 342.7
Vitamin K (0.01)	293.1	33.25	227.9 to 358.2
Cobalt (0.01)	289.1	29.24	231.8 to 346.4
Vitamin K (50)	284.7	35.1	215.9 to 353.5
Vitamin K (0.1)	282.2	56.15	172.1 to 392.2
Copper (100)	276.5	29.24	219.2 to 333.8
Vitamin D (0.01)	273.9	26.17	222.6 to 325.2
Cobalt (10)	271.9	42.28	189.1 to 354.8
Copper (1)	271.2	29.04	214.2 to 328.1
Zinc (0.01)	260.2	30.43	200.6 to 319.8
Vitamin E (100)	258.6	21.22	217.0 to 300.2
Vitamin B12 (0.1)	256.8	8.326	240.5 to 273.1
Zinc (50)	256.4	32.63	192.4 to 320.3
Vitamin K (10)	252.4	53.2	148.2 to 356.7
Vitamin E (0.01)	248.6	17.08	215.1 to 282.0
Vitamin B12 (500)	247.6	26.48	195.7 to 299.5
Copper (10)	246	28.16	190.8 to 301.2
Chromium (500)	244.7	64.92	117.5 to 371.9
Vitamin K (100)	244	29.41	186.4 to 301.6
Cobalt (100)	242.5	22.6	198.2 to 286.8
α-linolenic acid (0.1)	240.5	50.59	141.4 to 339.7
Zinc (10)	235.8	27.83	181.3 to 290.4
Cobalt (50)	233.2	16.94	200.0 to 266.4
Copper (0.1)	225.5	15.53	195.0 to 255.9
Vitamin E (10)	222.3	33.49	156.6 to 287.9
Chromium (0.01)	221	33.23	155.9 to 286.2
Vitamin K (1)	220.9	43	136.6 to 305.2

Zinc (100)	220.4	31.27	159.1 to 281.7
Vitamin D (1)	219	19.6	180.6 to 257.4
Iodine (0.1)	217.6	58.59	102.8 to 332.4
Thyroxine (0.1)	215.8	41.92	133.6 to 298.0
BR + TGFβ1	215.4	25.49	165.4 to 265.3
Vitamin D (0.1)	210.1	12.26	186.1 to 234.2
Zinc (1)	207.1	15.94	175.9 to 238.4
Vitamin D (10)	204.7	24.25	157.1 to 252.2
Vitamin A (0.01)	203.5	30.72	143.3 to 263.7
Zinc (0.1)	202	28.98	145.2 to 258.8
Manganese (0.1)	200	36.97	127.5 to 272.4
Manganese (50)	199	28.23	143.7 to 254.4
Vitamin E (1)	198	11.83	174.8 to 221.1
Cobalt (1)	197.1	36.02	126.5 to 267.7
Iodine (50)	196.8	50.85	97.16 to 296.5
α-linolenic acid (0.01)	190.1	41.5	108.7 to 271.4
Vitamin E (0.1)	189.2	16.26	157.3 to 221.0
Vitamin B7 (0.1)	187.7	48.11	93.42 to 282.0
α-linolenic acid (10)	186.2	10.96	164.8 to 207.7
Molybdenum (0.1)	185.1	41.19	104.4 to 265.8
α-linolenic acid (1)	185.1	15.99	153.8 to 216.5
Manganese (500)	183.7	42.03	101.3 to 266.0
Vitamin B7 (0.01)	181.3	27.8	126.8 to 235.8
Chromium (100)	180.7	12.43	156.4 to 205.1
Cobalt (0.1)	180.4	17.91	145.3 to 215.5
Vitamin E (50)	177.2	3.812	169.7 to 184.7
Manganese (0.01)	172.9	15.97	141.6 to 204.2
Copper (50)	167.7	13.26	141.7 to 193.7
Thyroxine (0.01)	167.1	62.27	45.00 to 289.1
Chromium (1)	162.1	6.33	149.7 to 174.5
Vitamin B7 (50)	159.6	16.71	126.9 to 192.4
Thyroxine (50)	158.5	12.43	134.2 to 182.9
Zinc (500)	156.7	52.09	54.57 to 258.7
Molybdenum (50)	156.5	16.05	125.0 to 187.9
Iodine (500)	155.7	14.64	127.0 to 184.4
Chromium (10)	155.3	34.66	87.38 to 223.2
Molybdenum (500)	155	29.99	96.24 to 213.8
Molybdenum (0.01)	154.2	15.7	123.5 to 185.0
Iodine (0.01)	152.8	10.71	131.8 to 173.8
Cobalt (500)	152.7	12.46	128.3 to 177.1
Manganese (1)	152.2	22.58	108.0 to 196.5
Thyroxine (1)	149.6	13.24	123.6 to 175.5
Vitamin B7 (1)	147.5	15.61	116.9 to 178.1

Manganese (10)	144.3	13.18	118.5 to 170.2
Vitamin B7 (500)	143.1	7.943	127.5 to 158.6
Thyroxine (10)	141.2	7.162	127.2 to 155.2
Iodine (10)	139.5	8.329	123.2 to 155.8
Vitamin D (50)	139.1	23.62	92.79 to 185.4
Molybdenum (1)	136.5	13.72	109.6 to 163.4
Iodine (100)	135.2	6.792	121.9 to 148.5
Manganese (100)	132.5	12.14	108.7 to 156.3
Vitamin B7 (10)	125.7	9.282	107.5 to 143.9
Vitamin B7 (100)	124.6	14.03	97.15 to 152.2
Molybdenum (10)	124.4	16.84	91.35 to 157.4
Molybdenum (100)	120.1	4.995	110.3 to 129.9
Iodine (1)	119.6	6.093	107.7 to 131.5
Thyroxine (100)	117.3	12.23	93.30 to 141.2
Thyroxine (500)	116.2	11.47	93.67 to 138.6
Vitamin E (500)	113.8	13.05	88.21 to 139.4
Vitamin D (100)	104.3	10.17	84.41 to 124.3
Vitamin D (500)	56.04	1.154	53.78 to 58.30
Copper (500)	54.15	4.816	44.71 to 63.59
Vitamin A (0.1)	53.1	5.784	41.76 to 64.44
Vitamin A (1)	38.77	12.79	13.70 to 63.84
α-linolenic acid (100)	38.66	1.248	36.22 to 41.11
Vitamin A (100)	38.49	15.45	8.204 to 68.78
Vitamin A (10)	36.95	7.876	21.51 to 52.38
Vitamin A (50)	16.57	1.479	13.67 to 19.46
Vitamin A (500)	12.13	0.9688	10.23 to 14.03
α-linolenic acid (500)	-14.43	0.5707	-15.55 to -13.31

Supplemental Data 8

The effect of vitamins and minerals on *Gaussia* luciferase luminescence was assessed by supplementation of conditioned medium with each of the vitamins and minerals tested. No significant changes were seen.

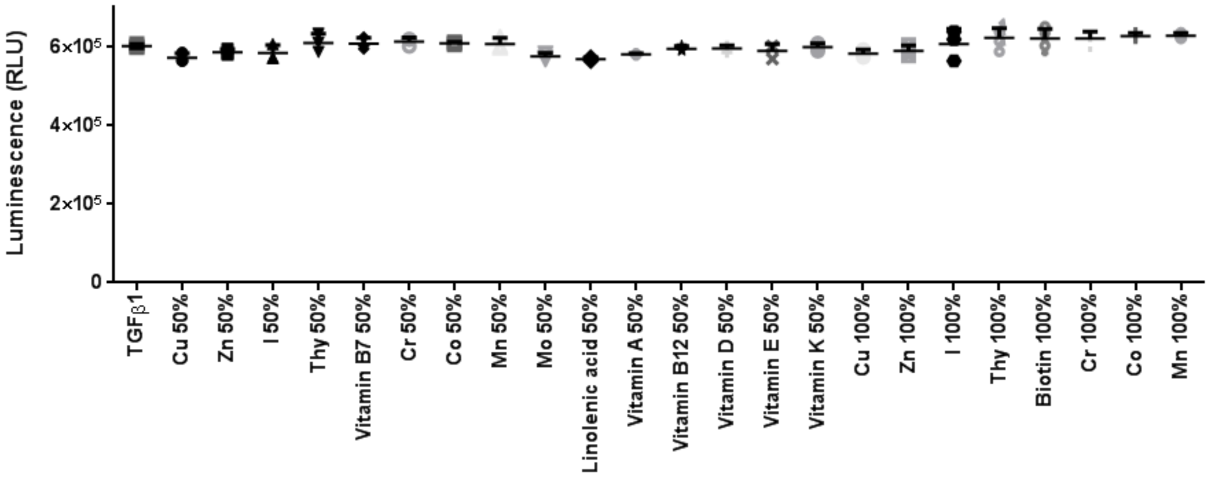

Supplemental Data 9

Plasmid maps for col2gluc (HPRM22364-LvPG02; Genecopoeia), the eGFP control (EX-EGFP-Lv105; GeneCopoeia), psPAX2 (plasmid # 12259; Addgene) and pMD2.G (plasmid # 12260; Addgene).

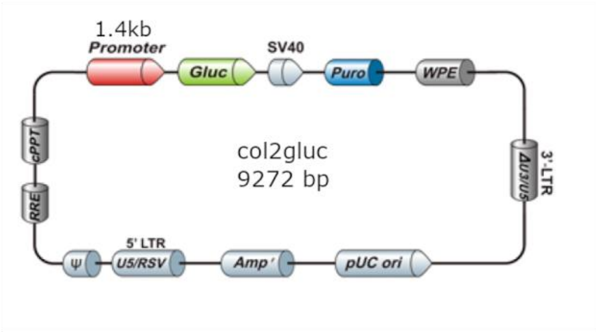

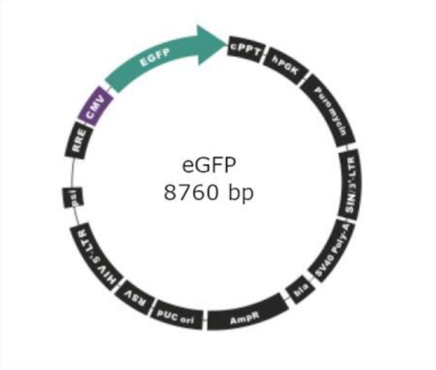

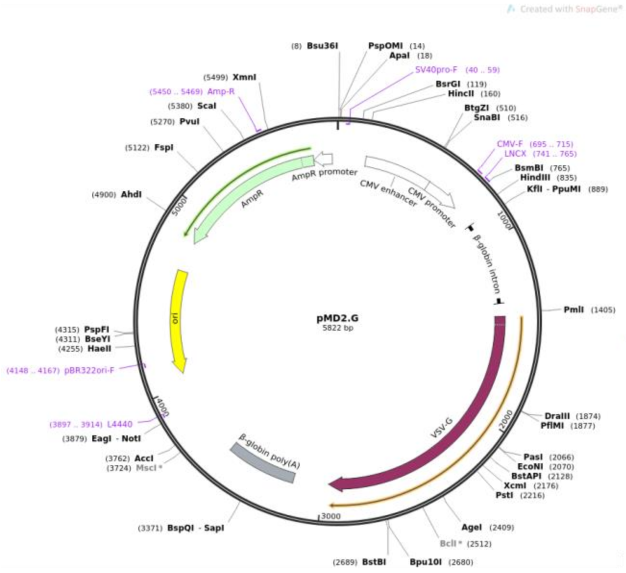

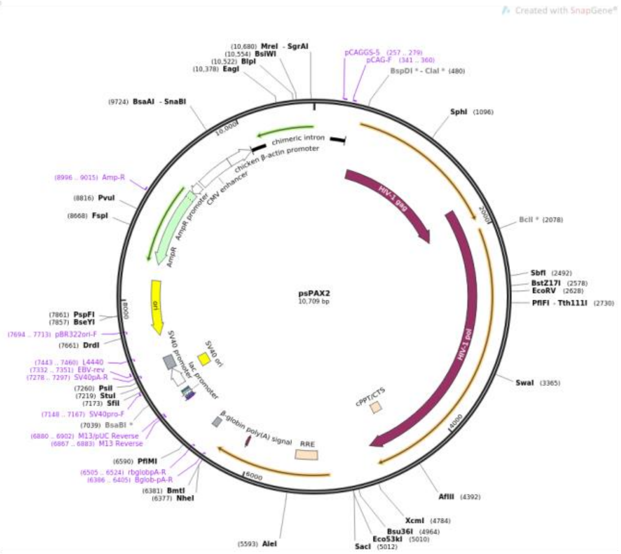

Supplemental Data 10

The following graphs all show type II collagen promoter-driven luciferase activity normalized to metabolic activity. Assessment was performed at day 21 following continuous stimulation with vitamins or minerals in the presence of TGFβ1 (1ng/ml) over a 5 log dose response. Conditions that were statistically greater than TGFβ1 supplemented medium are represented by a ▲ and an *; conditions that were similar to TGFβ1 supplemented medium are represented by a ●; conditions that were lower than TGFβ1 supplemented medium are represented by ▼; 2-way ANOVA with Dunnett's multiple comparison test, Alpha 0.05. A red dashed line indicates the mean TGFβ1 supplemented medium response.

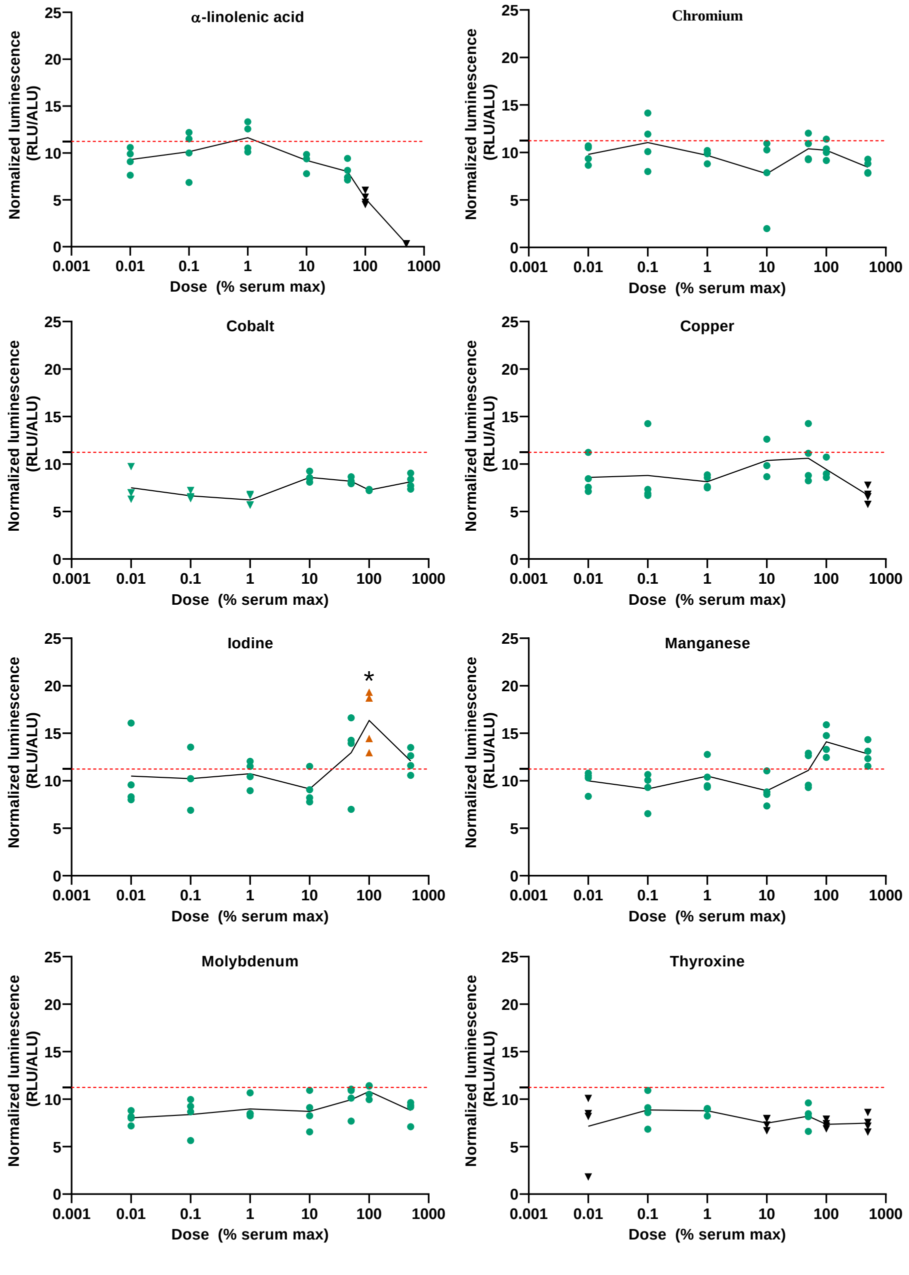

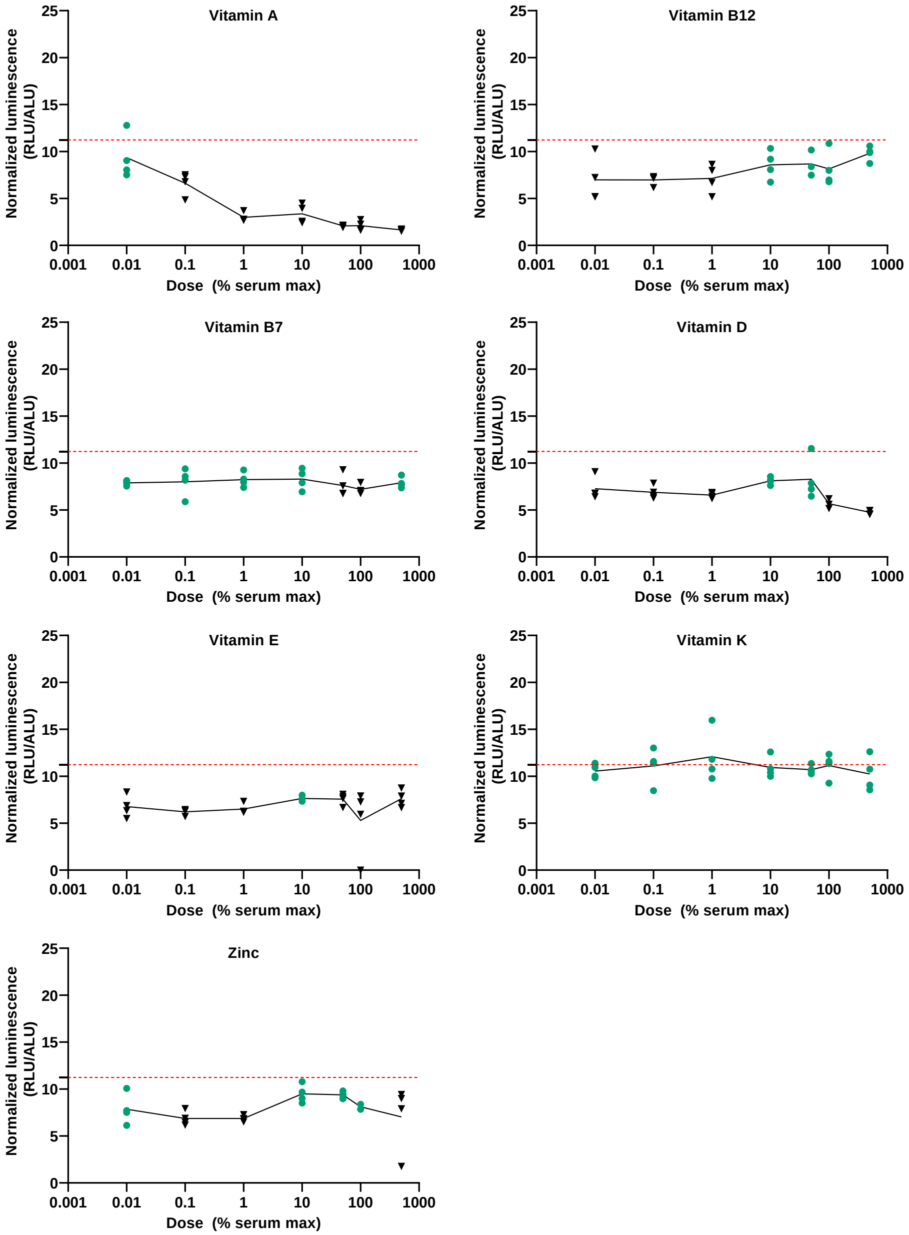
Vitamin A
Vitamin B12
Vitamin B7
Vitamin D
Vitamin E
Vitamin K
Zinc
Normalized luminescence (RLU/ALU)
Dose (% serum max)
0
5
10
15
20
25
0.001
0.01
0.1
1
10
100
1000
